# Genome and Transcriptome of *Clostridium phytofermentans*, Catalyst for the Direct Conversion of Plant Feedstocks to Fuels

**DOI:** 10.1371/journal.pone.0118285

**Published:** 2015-06-02

**Authors:** Elsa Petit, Maddalena V. Coppi, James C. Hayes, Andrew C. Tolonen, Thomas Warnick, William G. Latouf, Danielle Amisano, Amy Biddle, Supratim Mukherjee, Natalia Ivanova, Athanassios Lykidis, Miriam Land, Loren Hauser, Nikos Kyrpides, Bernard Henrissat, Joanne Lau, Danny J. Schnell, George M. Church, Susan B. Leschine, Jeffrey L. Blanchard

**Affiliations:** 1 Department of Microbiology, University of Massachusetts, Amherst, Massachusetts, United States of America; 2 Graduate Program in Molecular and Cellular Biology, University of Massachusetts, Amherst, Massachusetts, United States of America; 3 Institute for Cellular Engineering, University of Massachusetts, Amherst, Massachusetts, United States of America; 4 Commissariat à l'Energie Atomique et aux Energies Alternatives (CEA)-Genoscope, Unité Mixte de Recherche (UMR)-8030, National Center for Scientific Research (CNRS), Evry, France; 5 Department of Energy (DOE)- Joint Genome Institute, Genome Biology Program, Production Genomics Facility, Walnut Creek, California, United States of America; 6 Oak Ridge National Laboratory (ORNL), Life Sciences Division, Oak Ridge, Tennessee, United States of America; 7 Architecture et Fonction des Macromolécules Biologiques, Unité mixte de recherche (UMR)-6098, National Center for Scientific Research (CNRS), and Universités d’Aix-Marseille I and II, Marseille, France; 8 Department of Biochemistry and Molecular Biology, University of Massachusetts, Amherst, Massachusetts, United States of America; 9 Department of Genetics, Harvard Medical School, Boston, Massachusetts, United States of America; 10 Department of Veterinary and Animal Sciences, University of Massachusetts, Amherst, Massachusetts, United States of America; 11 Graduate Program in Organismal and Evolutionary Biology, University of Massachusetts, Amherst, Massachusetts, United States of America; 12 Department of Biology, University of Massachusetts, Amherst, Massachusetts, United States of America; Wageningen University, NETHERLANDS

## Abstract

*Clostridium phytofermentans* was isolated from forest soil and is distinguished by its capacity to directly ferment plant cell wall polysaccharides into ethanol as the primary product, suggesting that it possesses unusual catabolic pathways. The objective of the present study was to understand the molecular mechanisms of biomass conversion to ethanol in a single organism, *Clostridium phytofermentans*, by analyzing its complete genome and transcriptome during growth on plant carbohydrates. The saccharolytic versatility of *C*. *phytofermentans* is reflected in a diversity of genes encoding ATP-binding cassette sugar transporters and glycoside hydrolases, many of which may have been acquired through horizontal gene transfer. These genes are frequently organized as operons that may be controlled individually by the many transcriptional regulators identified in the genome. Preferential ethanol production may be due to high levels of expression of multiple ethanol dehydrogenases and additional pathways maximizing ethanol yield. The genome also encodes three different proteinaceous bacterial microcompartments with the capacity to compartmentalize pathways that divert fermentation intermediates to various products. These characteristics make *C*. *phytofermentans* an attractive resource for improving the efficiency and speed of biomass conversion to biofuels.

## Introduction

Plant biomass is one of the most abundant renewable energy sources on Earth and a largely underutilized feedstock for biofuels [1]. Production of biofuels from the lignocellulose fraction of plant biomass differs from production from grains in two fundamental aspects: (1) different types of saccharolytic enzymes are required to break down lignocellulose into soluble carbohydrates; and (2) fermentation of pentose sugars, in addition to hexoses, is required to harvest the majority of energy stored in lignocellulose [2]. At present, the cost of producing saccharolytic enzymes and the complexity of the hydrolysis and fermentation processes limit the use of plant biomass as a competitive alternative to gasoline and pose key challenges in the development of a global biomass industry for manufacturing a wide range of products from agricultural and forestry wastes [3].

One potential solution is the use of microbes that produce lignocellulose-decomposing enzymes and simultaneously ferment the resulting hexose and pentose carbohydrates to products such as ethanol. Merging these processes in a single microbe could substantially reduce the costs of lignocellulosic biofuel production [4]. Such microbes, primarily members of the *Clostridiales*, are found in natural anoxic environments where vast quantities of cellulose and other plant cell wall components are decomposed.

Species of *Clostridium* have a rich tradition in the development of biofuels. *Clostridium acetobutylicum* is a long-standing commercially valuable bacterium that has been used to produce acetone, butanol and ethanol from starch [5]. Processes based on acetone-butanol-ethanol fermentation were industry standards until the late 1940's, when low oil prices favored processes based on hydrocarbon cracking and petroleum distillation techniques. *C*. *acetobutylicum* and its relative *Clostridium beijerinckii* have recently regained market interest for use in the production of butanol as a gasoline and diesel fuel replacement.

Microbial fermentation of cellulose has been studied extensively in *Clostridium cellulolyticum* and *Clostridium thermocellum* [6–9]. Carbon metabolism during growth on cellulose and cellobiose in *C*. *cellulolyticum* has been investigated using carbon isotope labeling and metabolic flux analysis [8]. To degrade cellulose, *C*. *cellulolyticum* and *C*. *thermocellum* produce extracellular enzymatic complexes (cellulosomes) that permit bacterial adhesion to insoluble substrates and promote the hydrolysis of cellulose [6,9,10]. *C*. *acetobutylicum* laboratory strains do not grow on cellulose although they contain genes for cellulosome synthesis [7] and secrete a small cellulosome [11]. Products of cellulose degradation, such as cellobiose, are transported across the cell membrane and enter into the Embden-Meyerhof-Parnas pathway. *C*. *acetobutylicum* laboratory strains do not grow on cellulose although they contain genes for cellulosome synthesis [7] and secrete a small cellulosome [11].

We isolated a new species, *Clostridium phytofermentans* (strain ISDg ATCC 700394) from forest soil near the Quabbin Reservoir in Massachusetts, U.S.A. that directly ferments all major components of plant biomass, including cellulose, hemicellulose, pectin and starch to yield ethanol as the primary product of fermentation [12]. The combination of carbohydrate substrate versatility and high ethanol yield in a single organism distinguishes *C*. *phytofermentans* from other described species and suggests that it possesses unusual catabolic pathways. Extensive metabolism of the complex sugars within lignocellulose (without high ethanol yield) appears to be a trait found in several hyperthermophiles [13] but *C*. *phytofermentans* stands out as one of few, if not the only mesophile with this capacity. Thus, *C*. *phytofermentans* offers opportunities to understand the molecular mechanisms of plant biomass conversion to biofuels in a single organism. These attributes have led to the adoption of *C*. *phytofermentans* as a study system by groups in the US, Japan and Europe [14–25].

Here we investigate the unique properties of *C*. *phytofermentans* through analyses of its complete genome sequence and transcriptional profiling during growth on key components of plant biomass.

## Materials and Methods

### Growth and DNA extraction

*Clostridium phytofermentans* ISDg^T^ was cultured in anaerobic medium GS-2CB containing cellobiose (3 g/l) prepared as described previously [12]. Cultures were incubated in an atmosphere of O_2_-free N_2_ at 30°C. Genomic DNA was purified from 100 ml of mid exponential phase GS-2CB cultures using a standard DNA isolation procedure recommended by the Joint Genome institute, the Bacterial CTAB protocol [26].

### Construction, isolation and sequencing of insert libraries

Genomic DNA was sequenced using an established whole genome shotgun strategy [27]. Random 2–3 kb-DNA fragments were isolated after mechanical shearing. These gel-extracted fragments were concentrated, end-repaired and cloned into pUC18. Double-ended plasmid sequencing reactions were carried out using PE BigDye Terminator chemistry (Perkin Elmer) and sequencing ladders were resolved on PE 3700 Automated DNA Sequencers.

### Sequence assembly and gap closure

Sequence traces were processed with Phred [28] for base calling and assessment of data quality before assembly with Phrap [29] and visualization with Consed [30].

### Sequence analysis and annotation

Gene modeling was performed with both the Critica [31] and Glimmer [32] modeling packages. The results were combined and a basic local alignment search tool for proteins (BLASTP) versus GenBank's nonredundant database (NR) was conducted. The alignment of the N terminus of each gene model versus the best NR match was used to pick a preferred gene model. If no BLAST match was returned, the Critica model was retained. Gene models that overlapped by greater than 10% of their length were flagged, giving preference to genes with a BLAST match. In addition to BLASTP versus NR, the revised gene/protein set was searched against the KEGG GENES [33–35], InterPro [36] (incorporating Pfam [37], TIGRFams [38], SMART [39], PROSITE [40], PRINTS [41] and ProDom [42]) and Clusters of Orthologous Groups of proteins (COGs) [43] databases. From these results, functional categorizations were developed using the KEGG and COGs hierarchies. Initial criteria for automated functional assignment required a minimum 50% residue identity over 80% of the length of the match for BLASTP alignments, plus concurring evidence from the above profile methods (e.g. pfam). Putative assignments were made for identities down to 30%, over 80% of the length.

To determine whether *C*. *phytofermentans* produced cellulosomes, a position-specific amino acids matrix, based on the sequence of the cellulosome domains from the *Clostridium* species, *C*. *cellulolyticum*, *C*. *thermocellum* and *C*. *acetobutylicum*, was constructed and searched against a local database of predicted *C*. *phytofermentans* proteins using PSI-BLAST. In addition, we searched *C*. *phytofermentans* proteins using Pfam models of cohesins and dockerins. Analyses of the theoretical subcellular localization and signal peptide cleavage sites were carried out using PSORT (http://psort.hgc.jp/form.html). Transporters were annotated by TransAAP [44]. The complete sequence of *C*. *phytofermentans* was made available in August 2007 (accession number NC_010001).

### Phylogenetic analysis of 16S rRNA sequence

To elucidate the phylogenetic relationship between *C*. *phytofermentans* and other members of the class Clostridia, including non-sequenced genomes, 16S rRNA gene sequences of the isolate and closely related species were used for neighbor-joining analysis. Sequences were aligned using ClustalX Version 2 [45]. The phylogenetic tree was constructed by neighbor-joining in Phylip [46]. *Bacillus subtilis* was used as an outgroup. Bootstrap values were calculated using a heuristic search and 1,000 bootstrap pseudoreplications.

### Detection of carbohydrate-active enzymes in bacterial proteomes

The search for carbohydrate-active modules (glycoside hydrolases, glycosyltransferases, polysaccharide lyases and carbohydrate esterases) and their associated carbohydrate-binding modules (CBMs) in *C*. *phytofermentans* was performed exactly as for the daily updates of the Carbohydrate-Active enZYme (CAZy) database (http://www.cazy.org/). Briefly, the sequences of the proteins in CAZy were cut into their constitutive modules (catalytic modules, CBMs and other noncatalytic modules or domains of unknown function). The resulting fragments were assembled and formatted as a sequence library for BLAST [47] searches. Accordingly, each protein model from *C*. *phytofermentans* (and other bacterial proteomes) was searched via BLAST against the library of approximately 100,000 individual modules using a database size parameter identical to that of the NCBI nonredundant database. All models that gave an expectation value lower than 0.1 were automatically kept and manually analyzed. Manual analysis involved examination of the alignment of the model with the various members of each family (whether of catalytic or non-catalytic modules), with a search of the conserved signatures and motifs characteristic of each family. The presence of the catalytic machinery was verified for borderline cases whenever known in the family. The models that showed the usual features that would lead to their inclusion in the CAZy database were kept for annotation and classified in the appropriate class and family.

### Investigation of evolutionary origins of glycoside hydrolases

The taxonomic distribution of the BLASTP best hits (e-value< = 0.01) of the glycoside hydrolases in GenBank's nonredundant database (as of January 2011) excluding *C*. *phytofermentans* sequences was compared to that of BLASTP best hits of all the ORFs within the *C*. *phytofermentans* genome. A Pearson's Chi-squared test was used to determine the significance of the differences between the two taxonomic distributions.

### Culturing conditions for the microarray expression data

*C*. *phytofermentans* was cultured in a modified form of a previously described anaerobic medium [48] containing the following (g/l): yeast extract, 6.0; urea, 2.1; KH_2_PO4, 4.0; Na_2_HPO_4_, 6.5; trisodium citrate dihydrate, 3.0; L-cysteine hydrochloride monohydrate, 2.0; resazurin, 1; with pH adjusted to 7.0 using KOH. This medium was supplemented with 0.3% (wt/vol) of the following substrates: glucose, cellobiose, xylose, L-arabinose, birchwood xylan, and apple pectin (Sigma-Aldrich) as well as cellulose and “plant biomass” (*Brachypodium distachyon*). Substrates were added as a filter-sterilized solution to the sterile medium if soluble or autoclaved with the medium if insoluble. Duplicate liquid cultures were incubated at 30°C under anaerobic conditions (in an atmosphere of N_2_) as described by Hungate [49]. Growth on soluble substrates was determined spectrophotometrically by monitoring changes in optical density at 660 nm. Growth on solid substrates was estimated by visually monitoring and marking the reduction in biomass levels in the test tubes.

### RNA isolation

RNA was isolated from two replicates of each type of culture at mid exponential phase. Briefly, cells were flash-frozen by immersion in liquid nitrogen, harvested by centrifugation for 5 min at 8,000 rpm at 4°C and re-suspended in 100 μl in TE buffer pH 8 (EMD Chemicals) containing 2 mg/ml lysozyme (Sigma-Aldrich) and incubated at 37°C for 40 min. Total RNA was isolated using the RNeasy RNA purification kit (QIAGEN) according to the manufacturer’s instructions. Contaminating DNA in total RNA preparations was removed with RNase-free DNase I (QIAGEN).

### Determination of fermentation products

Non-gaseous fermentation products were determined by high-performance liquid chromatography **(**HPLC) and gas chromatography (GC). Acetate, ethanol, formate and lactate concentrations in culture supernatants were measured using a BioRad Aminex HPX 87H 300 x 7.8 mm column at 55°C with 0.005 M H_2_SO_4_ as the mobile phase and a flow rate of 0.60 ml/min, in a Hitachi model L-7100 HPLC unit equipped with a Sonntek Refractive Index Detector. The concentration of ethanol was also measured by GC, using a Shimadzu GC 2014 with a Flame Ionization Detector and a Restek stabilwax-DA 30 m x 0.25 mm ID column (film thickness 0.25 μm). The carrier gas was helium at a flow rate of 1.5 ml/min. Injector and detector temperatures were both 200°C and the column temperature began at 70°C for 2 min, ramped to 175°C at 20°C/min, and was held at 175°C for 2 min.

### Microarray design

A *C*. *phytofermentans* Affymetrix microarray was custom-designed for the measurement of the expression level of all open reading frames, estimation of the 5’ and 3’ untranslated regions of mRNA, operon determination, and discrimination between alternative gene models (differing primarily in the selection of the start codon). Putative protein coding sequences were identified using both GeneMark and Glimmer, and the union of these two predictions was used to design the array. Each coding sequence (CDS) was represented by eleven 24-mer probes. Standard Affymetrix array design protocols were followed to ensure each probe was unique to minimize cross hybridization. If two CDS differed only in their N-terminal region, the smaller of the two proteins was used for transcript analysis, but the extended region was also represented by probes to define the actual N-terminus. Remaining probes were used to map expression in intergenic regions. These probes represented both DNA strands and were tiled with a 1-nucleotide gap. The array design was implemented on a 49–5241 format Affymetrix GeneChip with 11-μm features. The microarray was designed prior to the final annotation of the complete genome using gene prediction methods that are slightly different from those used in the final annotation done by the Joint Genome Institute. The microarray files uploaded to NCBI's GEO reflect the later complete genome annotation done by the Joint Genome Institute.

### Microarray processing

cDNA synthesis, array hybridization and imaging were performed at the Genomic Core Facility at the University of Massachusetts Medical Center. Ten μg total RNA from each sample was used as template to synthesize labeled cDNAs using Affymetrix GeneChip DNA Labeling Reagent Kits. The labeled cDNA samples were hybridized on the arrays according to Affymetrix guidelines. The hybridized arrays were scanned with a GeneChip Scanner 3000. The resulting raw spot image data files were processed into pivot, quality report, and normalized probe intensity files using Microarray Suite version 5.0 (MAS 5.0) [50]. In addition, expression values were calculated using the Custom Array Analysis Software (CAAS) package (http://www.sourceforge.net/projects/caas-microarray/) that implements the Robust Multichip Average method [51]. The individual microarray files and the normalized gene summary values for the complete data set will be deposited in the Gene Expression Omnibus (GEO) database at NCBI [52].

The quality of the microarray datasets were analyzed using probe-level modeling procedures provided by the affyPLM package in BioConductor [52]. No image artifacts due to array manufacturing or processing were observed. Microarray backgrounds were within the typical 20–100 average background values for Affymetrix GeneChip. In summary, all quality control checks indicated that the RNA purification, cDNA synthesis, labeling and hybridization procedures adapted for use in *C*. *phytofermentans* resulted in high quality data. All microarray data reported in the text and figures represent the average of expression values derived from two independent RNA preparations from duplicate cultures.

## Results and Discussion

**Fig 1:**
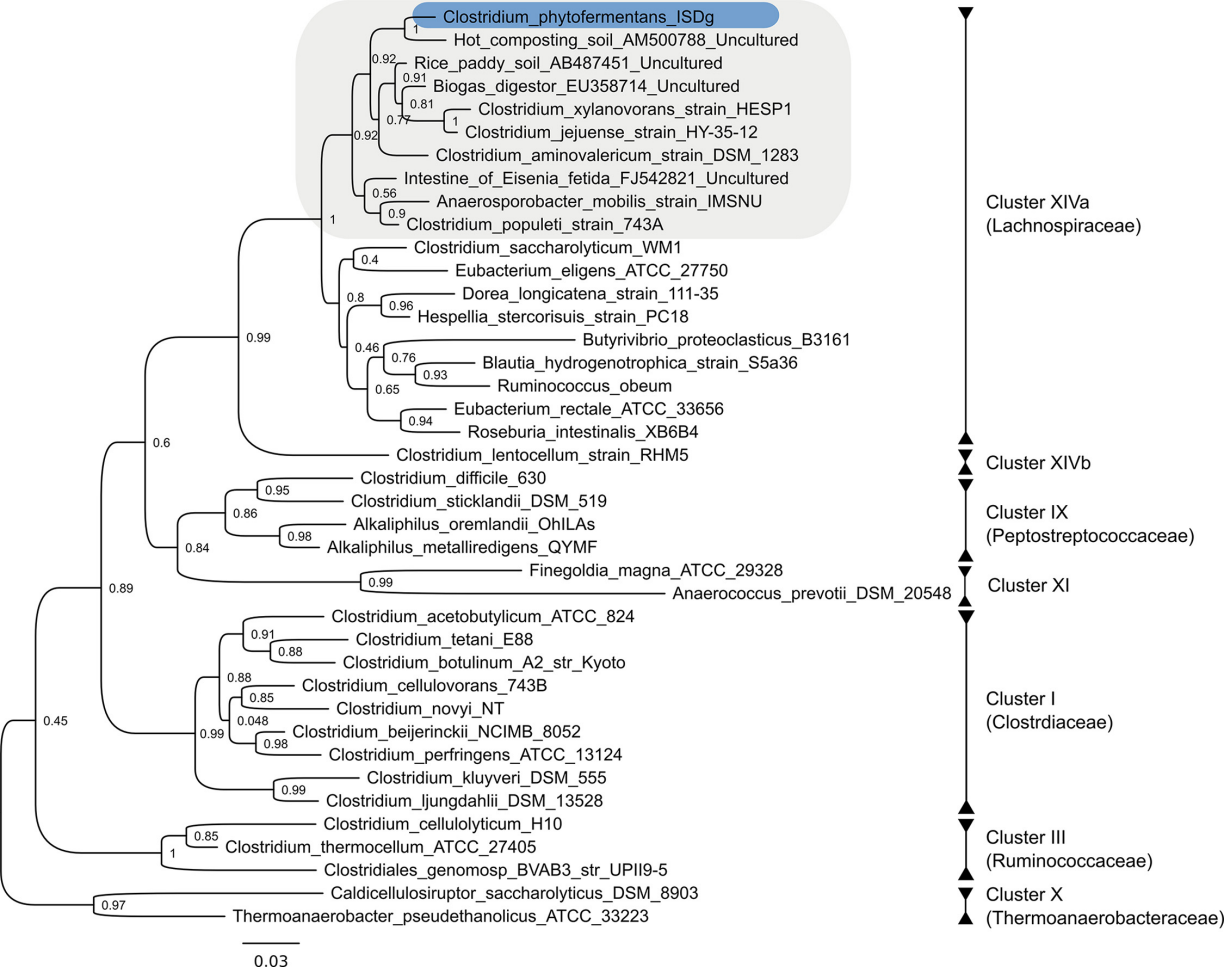
Neighbor-joining tree of *C*. *phytofermentans* and related taxa within the class Clostridia based on 16S rRNA gene sequences. Taxa with sequenced genomes are marked with an asterisk. Cluster numbers correspond to the cluster system of Collins et al. [68]. Bootstrap values were determined for 1,000 replicates.

*C*. *phytofermentans* is distinct from other well-studied solventogenic and cellulolytic species found within clostridial Clusters I (Clostridiaceae), III (Ruminococcaceae), and X (Thermoanaerobacteraceae) (Fig 1). A member of Cluster XIV (Lachnospiraceae), *C*. *phytofermentans* is closely related to human commensals that have been sequenced as part of the International Human Microbiome Consortium [53], and to bacteria isolated from rice paddy soils, earthworm intestines and other anaerobic, carbon rich environments (Fig 1). As a genetically tractable [15] member of this under-explored group, and the first with a publicly available genome sequence, *C*. *phytofermentans* is an important point of reference for comparative genomic analyses.

### Features of the *C*. *phytofermentans* genome

*C*. *phytofermentans* has a single circular 4.8 Mbp chromosome, no plasmids and a G+C content of 35%. The genome encodes 3,926 CDS, 27% of which lack a predicted function (Table 1). Genes encoding eight rRNA clusters were found in proximity to the origin of replication and 61 tRNAs were detected (Table 1). Two putative prophage regions were found (S1 File). Genes for processes typical of clostridia, such as sporulation (S2 File), motility and chemotaxis, are present. Identification of sporulation-related genes is typically based on sequence homology to those of *Bacillus subtilis–*the model organism for studying the sporulation cycle [54–56]. The genome of *C*. *phytofermentans* contains a homolog of the master regulator of sporulation of *B*. *subtilis*, SpoOA (Cphy_2497, 55% amino acid identity to SpoOA of *B*. *subtilis*, Table A in S2 File). Although the majority of the genes in the sporulation cascade of *B*. *subtilis* downstream of the master regulator SpoOA are present in *C*. *phytofermentans*, the ones upstream (the sensory histidine kinase and phosphorelay system) are not. *C*. *phytofermentans* is motile, moving by means of one or a few sub-terminal flagella [12]. Genes predicted to be involved in flagellar biosynthesis are found in two clusters (Cphy_0303–0316 and Cphy_2687–2720). Chemotaxis genes are found within one of these clusters (Cphy_2687–2691). *C*. *phytofermentans* also has three distinct genetic loci coding for proteinaceous bacterial microcompartments [57,58] (S3 File), two of which are predicted to be involved in choline, ethanolamine and 1,2-propanediol metabolism and one whose function cannot be inferred from sequence analysis.

**Table 1:** General features of the genome of *C*. *phytofermentans*.

Parameter	Value
Size (bp)	4,847,594
G+C content (%)	35
Protein coding genes	
No. similar to known proteins (%)	2,870 (73.1)
No. similar to proteins of unknown function ^a^ (%)	170 (4.3)
No. of conserved hypotheticals ^b^ (%)	265 (6.7)
No. of hypotheticals ^c^ (%)	621 (15.8)
Total	3,926
Average ORF size (bp)	1,009
Coding (%)	81
No. of rRNA clusters	8
No. of tRNA genes	61

^a^ Unknown function indicates significant sequence similarity to a named protein to which no specific function is currently attributed.

^b^ Conserved hypothetical proteins share significant sequence similarity to a translation of an open reading frame (ORF) in another organism for which no experimental evidence of protein expression is not available.

^c^ Hypothetical proteins with no significant similarity to any other sequenced gene.

### Genes encoding carbohydrate-active enzymes

**Fig 2:**
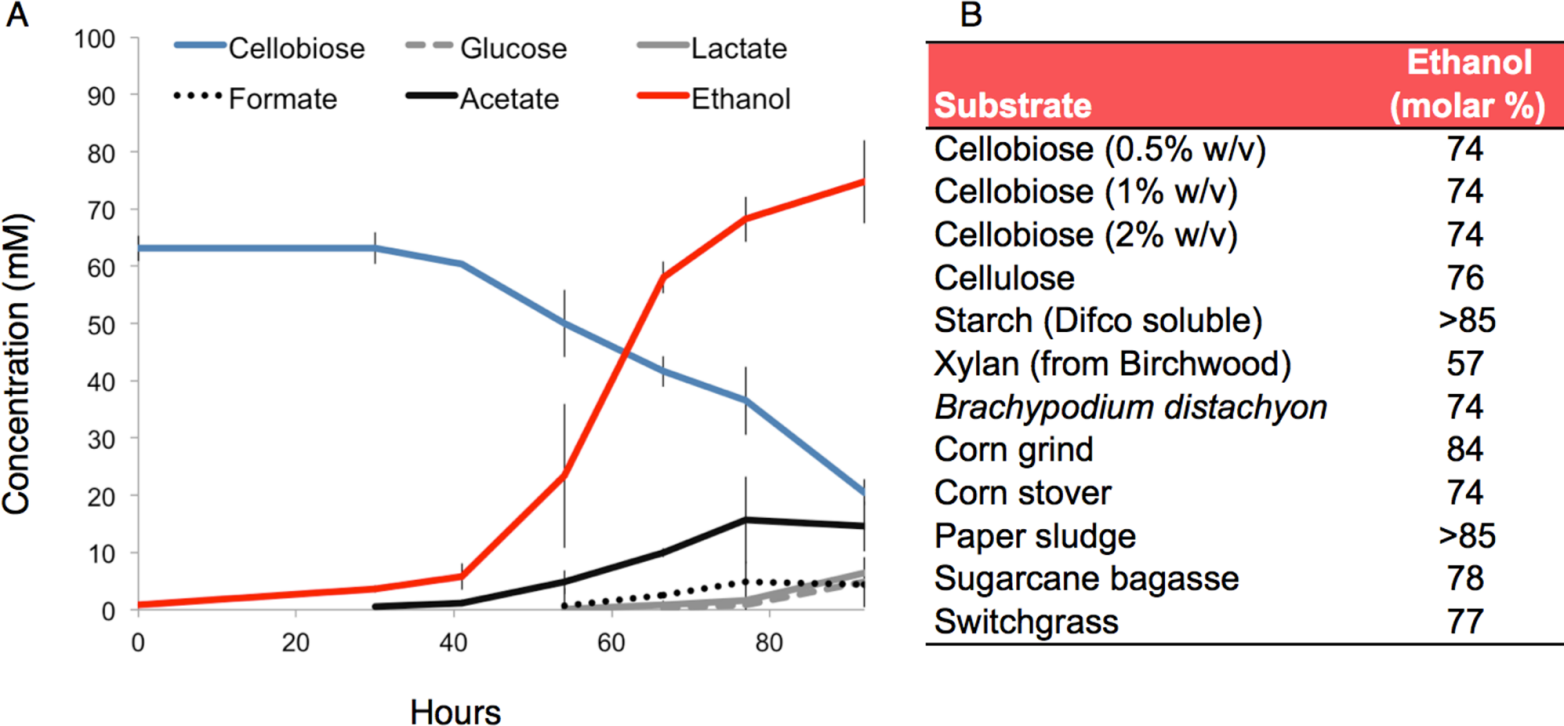
Fermentation products on different growth substrates. **(A)** Fermentation products during growth on 2% (w/v) cellobiose. Data are an average of two samples; error bars represent range. **(B)** Ethanol produced on a variety of substrates expressed as the molar percentage of non-gaseous products. All substrates were present at a concentration of 1% (w/v) except where otherwise indicated. The particle size of insoluble substrates was reduced by grinding; the substrates were not otherwise pre-treated. Fermentation products were measured after obvious growth ceased (3–5 days) at 30°C. In most cases, substrate conversion was incomplete.

**Fig 3:**
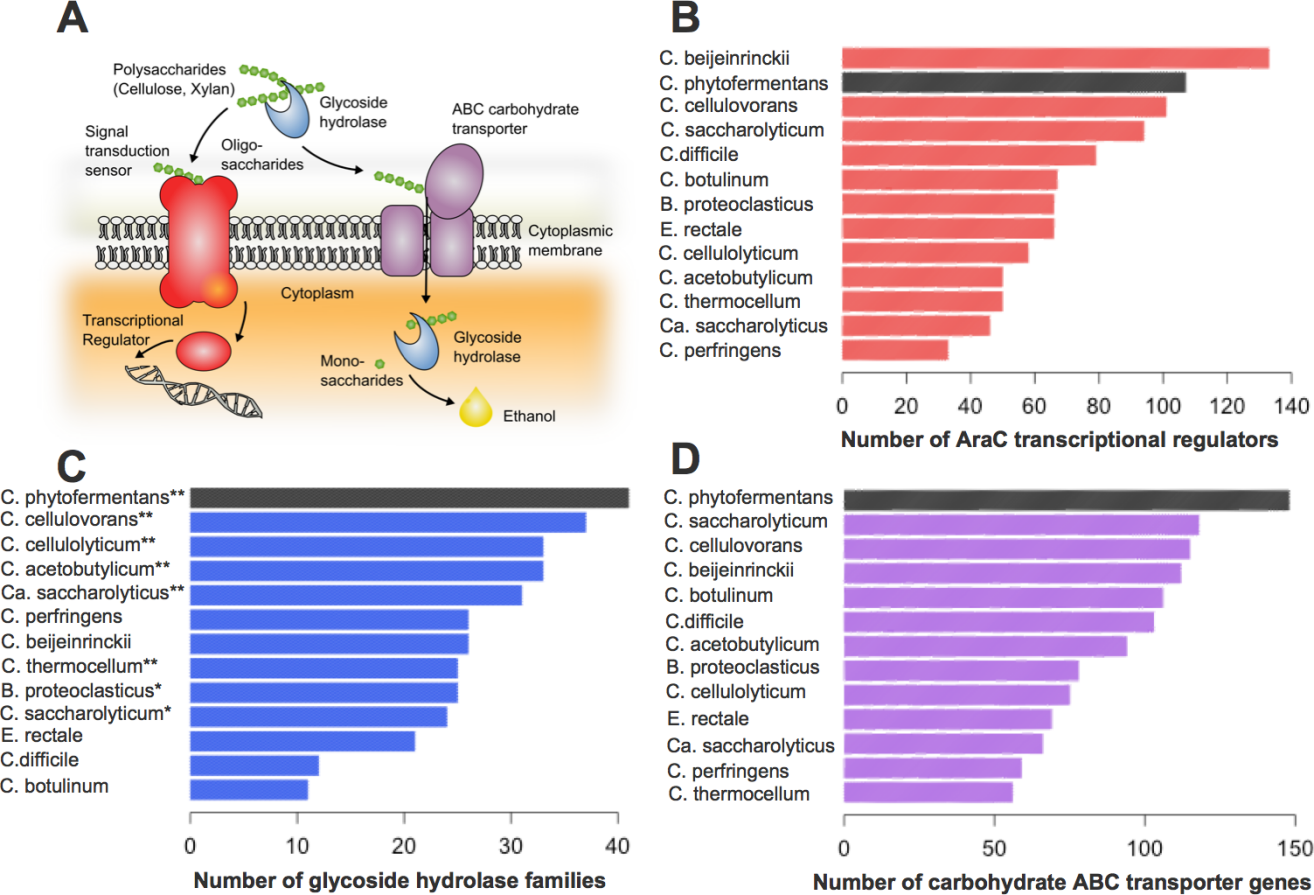
Comparative analysis of AraC transcriptional regulators, glycoside hydrolases (GH), and ABC transporters among selected sequenced clostridial genomes. **(A)** A conceptual illustration of how GH (blue), ABC transporters (purple) and AraC regulators (red) may work together. **(B)** Number of AraC transcriptional regulators per genome. **(C)** Number of GH domains per genome. Organisms having both GH48 and GH9 are marked with two asterisks, and organisms having GH9 alone are marked with one asterisk. **(D)** Number of putative ABC transporters per genome.

*C*. *phytofermentans* is capable of breaking down the recalcitrant, insoluble components of plant cell walls including cellulose, hemicellulose, pectin and starch [12] as well as switchgrass, corn stover and pulp wastes that have been minimally processed without thermo-chemical pretreatment (Fig 2). Numerous carbohydrate-active enzymes (CAZy) predicted to be involved in the degradation of various plant cell wall components are encoded throughout the *C*. *phytofermentans* genome, including glycoside hydrolases (GH), polysaccharide lyases (PL) and carbohydrate esterases (CE). The diversity of GH families in the *C*. *phytofermentans* genome is unparalleled among sequenced clostridial genomes (Fig 3). A total of 116 GHs distributed among 44 families are encoded in the genome of *C*. *phytofermentans* including but not limited to endo- and exo-cellulases, hemicellulases, chitinases, pectinases, amylases, and lichenases (Fig 3 and S4 File). Only the GH content of a distant relative in Cluster I, *Clostridium cellulovorans*, is comparable, with 113 GH domains distributed among 37 families (Fig 3). A closer relative of *C*. *phytofermentans*, *Butyrivibrio proteoclasticus* (Fig 3), has a comparable number of GH domains (113), but less diversity with only 25 families and no exo-cellulase (GH48).

**Fig 4:**
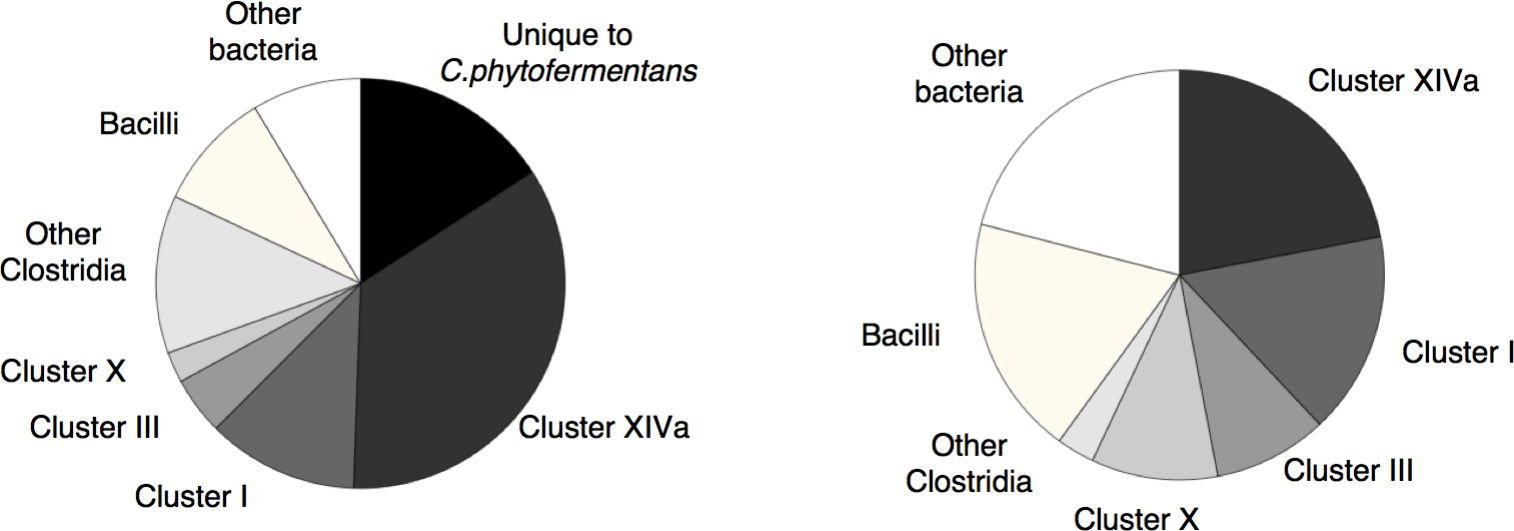
Comparison of the distribution of the closest relatives of all *C*. *phytofermentans* open reading frames among sequenced bacterial genomes (left) to that of closest relatives of its glycoside hydrolases (right).

To gain insight into the origin of the GHs of *C*. *phytofermentans*, we identified the closest relatives of the GHs of *C*. *phytofermentans* in the GenBank database using BLASTP and compared their distribution to that of the closest relatives of all of the protein-coding genes within the *C*. *phytofermentans* genome. The latter analysis was performed to calibrate how much similarity to other bacteria would be expected on average. In total, approximately 40% of the GHs of *C*. *phytofermentans* were most similar to GHs present in species outside the class Clostridia (Fig 4), whereas only 20% of all the genes in the *C*. *phytofermentans* genome were most similar to genes from outside the Clostridia. The higher than expected proportion of GHs with distant relatives is statistically significant (Pearson's Chi-squared test, X-squared = 77.8583, df = 9, p-value = 4.299e-13) (Fig 4). This result suggests that horizontal gene transfer from diverse origins rather than vertical divergence from an ancestral genome played a key role in the assembly of the unique set GHs present in *C*. *phytofermentans*.

In some bacteria, notably *C*. *cellulolyticum* and *C*. *thermocellum*, lignocellulose-degrading enzymes are attached to complex extracellular structures called cellulosomes that are believed to be critical for efficient plant cell wall breakdown. However, there is no genomic evidence for the production of cellulosomes by *C*. *phytofermentans* (S4 File). In fact, two critical cellulases of *C*. *phytofermentans*, the GH9 family endocellulase and GH48 family exocellulase are more similar to the soluble cellulases of *C*. *thermocellum* than to cellulosomal cellulases [15]. The majority of the GHs of *C*. *phytofermentans* are multimodular. Carbohydrate-binding modules (CBMs) are found within 17% of the GHs of *C*. *phytofermentans*, including the critical endocellulase (GH9) [15]. In the absence of a cellulosome, these CBM domains may enable GHs to adhere to plant cell wall substrates, facilitating degradation of the heterogeneous, highly cross-linked lignocellulose polysaccharides. Among the 31 GH enzymes predicted to be extracellular, 16 contain domains involved in anchoring proteins to the cell surface, including transmembrane helices and/or cell-wall binding domains, suggesting that these enzymes are cell-associated. Despite the absence of cellulosomal assembly domains, the striking multimodular nature of cellulosomal proteins, in which multiple domains from diverse families of GH, CE, PL and carbohydrate-binding modules (CBM) are found within individual proteins, is preserved in *C*. *phytofermentans* (Table A in S4 File). *C*. *phytofermentans* has 19 multimodular GH proteins, representing about 17% of all putative GH genes (Table A in S4 File). In fact, the largest protein in the proteome is the multimodular glycoside hydrolase family 10 protein Cphy_3862, with 2457 amino acids and a predicted molecular weight of 266 kD [16]. This protein contains consecutive GH10, CE15, and CBM domains. In non-cellulolytic bacteria, the corresponding GH domains are found mainly in single-domain polypeptides, which are cytosolic and act on smaller, soluble carbohydrate substrates [59]. Thus, the multi-modular organization that seems to be characteristic of enzymes from cellulolytic species, may reflect their involvement in the extracellular processing of heterogeneous insoluble substrates, such as plant cell walls [59]. Biofilm formation may also play an important role in the orchestration of the degradation of the plant cell wall polysaccharides. Cells might adhere to each other via a variety of different domains such as pfam07705 (CARDB, cell adhesion domain in bacteria) and pfam01391 (Collagen, Collagen triple helix repeat), both of which are found in the *C*. *phytofermentans* genome.

### Genes potentially involved in carbohydrate transport

Further examination of the genome revealed 148 genes encoding subunits of ATP-binding cassette (ABC) transporters, more than found in other clostridia (Fig 3). These genes are typically organized in operons consisting of two permeases and one solute-binding component. The majority of the ABC transporter-encoding operons lack an ATPase, suggesting that these transporter complexes may interact with a multitasking ATPase. Cphy_3611 is similar to MsmX of *Bacillus subtilis*, which is proposed to be an ATPase for several oligosaccharide transporters [60]. These findings suggest that *C*. *phytofermentans* is capable of active uptake of a diverse array of metabolites, including multiple oligosaccharides and simple sugars. The presence of GH genes adjacent to 50% of the transporter loci, suggests that carbohydrate degradation and uptake are frequently coupled. *C*. *phytofermentans* may feed cytoplasmic oligosaccharides into glycolysis via cellobiose/cellodextrin phosphorylases as occurs in other cellulolytic bacteria [8]. Import of oligosaccharides followed by internal hydrolysis via phosphorolysis minimizes ATP consumption [3].

### Genes potentially involved in the regulation of carbohydrate metabolism

To orchestrate the regulation of diverse metabolic pathways in response to changing growth substrates, *C*. *phytofermentans* has numerous transcriptional regulators, including 70 AraC (Fig 3) and 23 PurR family members. AraC regulators typically activate transcription of genes involved in carbon metabolism, stress responses and pathogenesis [61], whereas PurR regulators act as repressors [62]. The abundance of these regulators suggests a complex regulatory network allowing rapid adaptation to varying substrate availability. Among the ABC-transporter genes found clustered with GHs, 50% are adjacent to AraC and 25% to PurR regulator genes.

### Analysis of gene expression during growth on a variety of simple and complex carbohydrates

**Fig 5:**
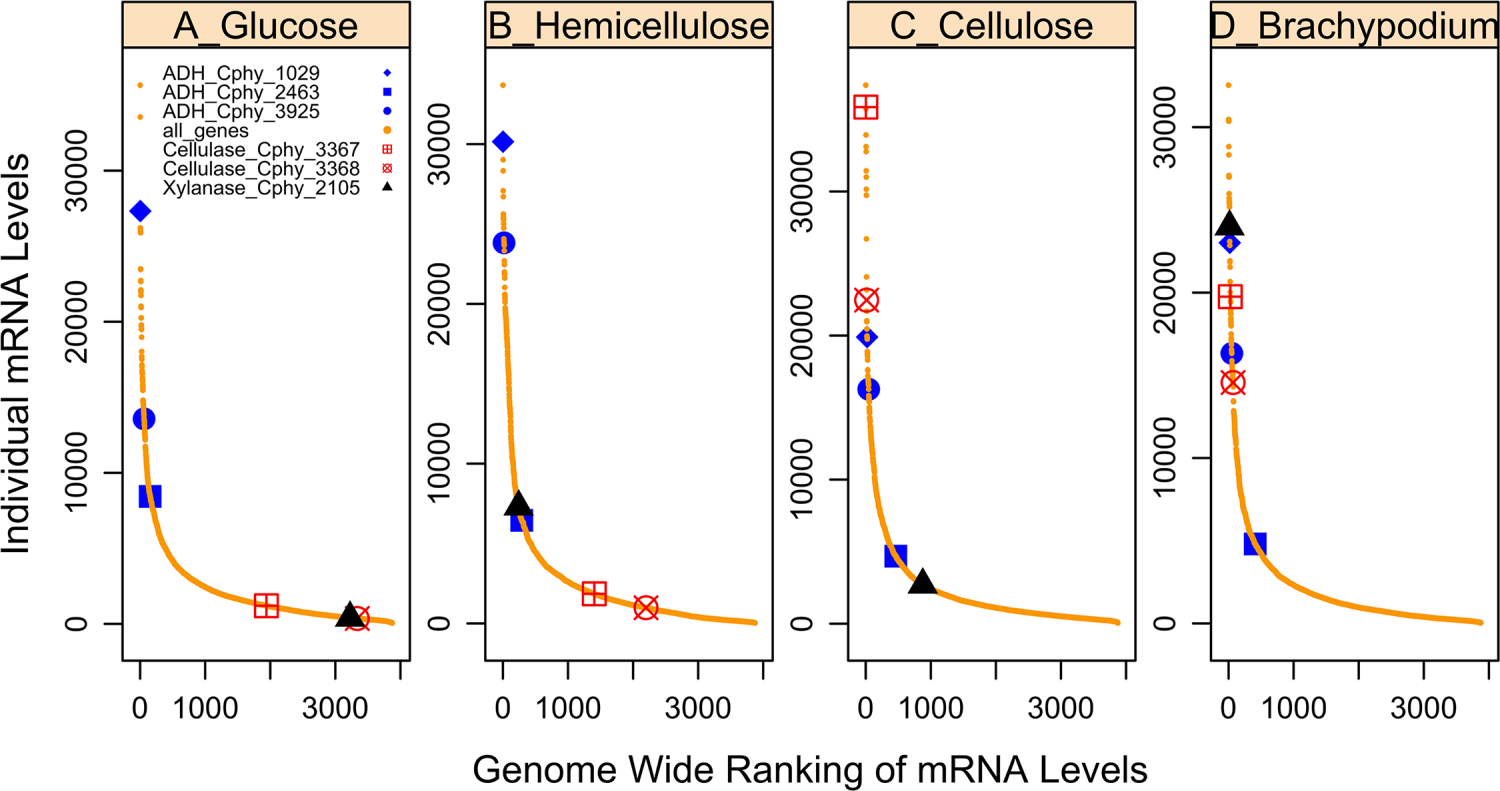
Illustration of the variation in transcription level of selected genes on various substrates. Transcript rank abundance curves during growth on **(A)** glucose, **(B)** hemicellulose, **(C)** cellulose and **(D)** Brachypodium. ADH_Cphy_1029 refers to a putative alcohol dehydrogenase. Cellulase_Cphy_3368 denotes the putative cellulose. Xylanase_Cphy_2105 denotes the putative xylanase.

We designed a custom Affymetrix GeneChip to identify genes expressed in *C*. *phytofermentans* during growth on monosaccharides that are common in plant cell walls (glucose, galactose, xylose, arabinose, mannose), purified polysaccharides (cellobiose, cellulose, xylan and pectin) and fibrous plant biomass (*Brachypodium distachyon*) (S4 File and S6 File). These microarray studies suggest that *C*. *phytofermentans* regulates the stoichiometry of the plant degradative and assimilatory machinery in response to growth substrate. When *C*. *phytofermentans* was cultured with glucose, genes involved in biomass degradation (e.g. cellulase and xylanase) were essentially off (Fig 5), and the most abundant transcript was a putative ABC monosaccharide transporter (S6 File). During growth on xylose and xylan, transcripts for enzymes involved in pentose interconversion (xylose isomerase (Cphy_0200, and Cphy_1219) and xylulokinase (Cphy_3419) were among the most highly expressed (S6 File). When *C*. *phytofermentans* was grown with cellulose as substrate, the GH9 cellulase gene was among the most abundant transcripts (Fig 5, cellulase_Cphy_3368). This cellulase gene has been shown by gene inactivation to be essential for growth on cellulose in *C*. *phytofermentans* [15]. On nearly all substrates tested, we observed specific sets of co-regulated groups of genes, often consisting of GHs, an ABC transporter and a transcriptional regulator (Tables C and D in S4 File and S5 File). The putative multitasking ABC transporter ATPase subunit Cphy_3611, was expressed during growth on all substrates (transcript abundance within the 50^th^ percentile) (S5 File). To orchestrate the regulation of these genes, a number of transcriptional regulators, typically physically close to the transporters and CAZy, are highly expressed on a given substrate (Tables C and D in S4 File). Thus, microarray experiments facilitated identification of enzymes involved in the breakdown and transport of specific carbohydrates. In addition, expression profiling with defined substrates was useful for deciphering data from more complex fibrous substrates. When plant biomass was used as growth substrates, GH expression profiles were similar to each other and to profiles with cellulose as substrate, with the exception that a putative xylanase (Cphy_2105) and mannanase (Cphy_1071) were more highly expressed on the plant biomass than on cellulose or xylan (S4 File). Thus, gene expression analysis proved to be a useful strategy for deciphering the functions of diverse enzymes involved in lignocellulose degradation (Fig 5, S4 File).

### Genomics and transcriptomics investigation of *C*. *phytofermentans* central metabolism

**Fig 6:**
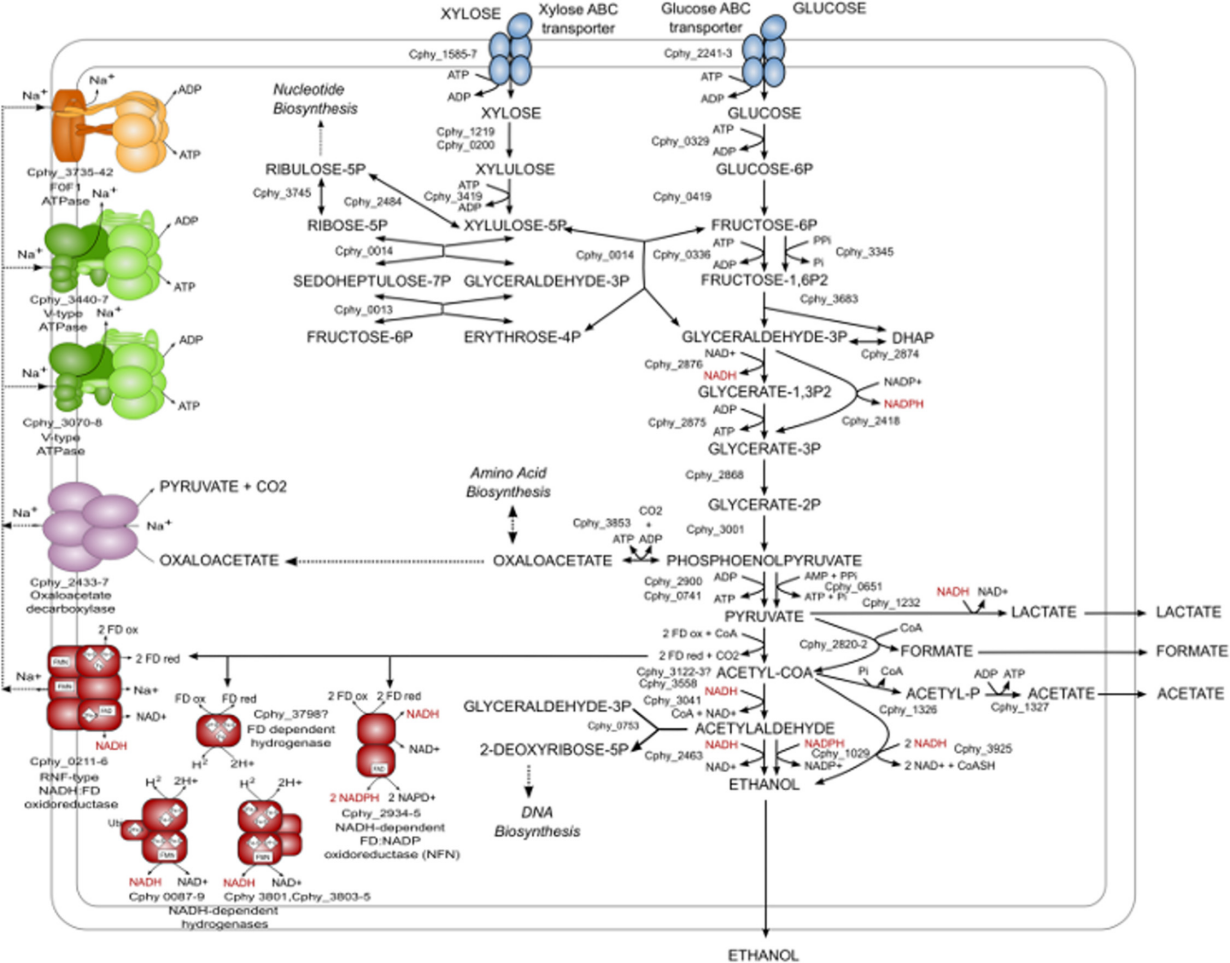
Model of *C*. *phytofermentans* central metabolism including proposed pathways involved in high ethanol yield.

Perhaps the most industrially relevant property of *C*. *phytofermentans* is that it produces ethanol as the major fermentation product during growth on a wide variety of substrates including, simple sugars, cellulose and minimally processed plant biomass [12] (Fig 2). The fact that *C*. *phytofermentans* produces predominantly ethanol, suggests that it can maintain its redox balance without forming equivalent levels of lactate and/or formate and that it can generate sufficient energy for growth in the absence of high levels of acetate synthesis, which yields ATP via substrate-level phosphorylation. To gain insight into the basis for high levels of ethanol production, we used a combination of transcriptional profiling and comparative genomic analysis to identify a subset of genes that were both highly expressed on all growth substrates and predicted to be involved in ethanol production, energy conservation, and/or redox balance. The results of this analysis are the basis of a simplified model of the core physiology of *C*. *phytofermentans* (Fig 6, S7 File) and indicate that high levels of ethanol production may be due to a combination of factors. Firstly, pyruvate appears to be funneled to ethanol. The levels of the transcripts of the enzymes within the ethanol biosynthesis pathway are extremely high, and exceed those of all enzymes involved in the synthesis of alternate carbon fermentation products (Fig 5, Table A in S7 File). In particular, two alcohol dehydrogenases (ADH), Cphy_3925 and Cphy_1029, were constitutively transcribed at levels that rivaled or exceeded those of many ribosomal protein genes (average transcript abundances within the 98th percentile, Fig 5 and Table A in S7 File). Examination of these genes revealed that both NADH and NADPH are likely to contribute to ethanol production, another factor that may increase ethanol production. Secondly, reduced ferredoxin generated during conversion of pyruvate to the ethanol precursor, acetyl-CoA, by pyruvate ferredoxin oxidoreductase may contribute to ethanol production both directly, through reduction of NAD and NADP and indirectly, by participating in energy conservation. Two constitutively highly expressed protein complexes are likely to play a role in enabling reduced ferredoxin to contribute to ethanol production: NfnAB, an NADH-dependent reduced ferredoxin:NADP oxidoreductase [63] and Rnf, a sodium-translocating NADH:ferredoxin oxidoreductase [64]. *C*. *phytofermentans* may be able to exploit the sodium gradient produced by Rnf for energy production by way of the highly expressed sodium-translocating F_1_F_o_-ATPase (Cphy_3735–42). Thus, Rnf may contribute to favorable energetics of ethanol production and reduce dependence on ATP generation via acetate production. Hydrogenases may also play an important role in ferredoxin metabolism in *C*. *phytofermentans*. In other clostridia, hydrogenases dissipate excess ferredoxin-reducing equivalents [65,66]. *C*. *phytofermentans* generates free hydrogen as a product of the fermentation of cellulose and cellobiose [67], and three cytoplasmic [FeFe]-hydrogenase-encoding clusters, one encoding a putative ferredoxin-dependent hydrogenase and two encoding NAD-dependent hydrogenases were constitutively highly expressed. The simultaneous expression of a ferredoxin-oxidizing hydrogenase with NAD-dependent hydrogenases, which could catalyze hydrogen-dependent NAD reduction and feed ferredoxin reducing equivalents into ethanol production, may prevent excess hydrogen accumulation thus enabling *C*. *phytofermentans* to maintain a high rate of ferredoxin turnover. Finally, *C*. *phytofermentans* may further reduce the requirement for acetate production by utilizing pyrophosphate–dependent glycolytic enzymes, which can substantially increase the ATP yield of glycolysis.

## Conclusions

Analysis of the *C*. *phytofermentans* genome revealed a diverse array of genes for metabolism of lignocellulosic biomass and production of alcohols and hydrogen that constitute a unique repertoire among sequenced clostridial genomes with relevance for the biofuels industry. Our analysis of the genome revealed the genomic basis for the generalist behavior of this microbe. The diverse CAZy likely enable complete hydrolysis of cellulosic and hemicellulosic substrates. Unexpectedly, we found no evidence of cellulosomes in *C*. *phytofermentans*, suggesting that *C*. *phytofermentans* has evolved alternative strategies to optimize degradation and uptake of plant cell wall components [16]. The absence of a cellulosome simplifies strategies for engineering levels of individual enzymes to improve the conversion of plant biomass to fermentable sugars. Many active sugar transporters are in close proximity to polysaccharide hydrolases, likely cooperating for efficient simultaneous degradation and uptake of carbohydrate growth substrates. Further investigation will be required to determine the substrate-specificity of these transporters.

Genomic analysis and transcriptional profiling also suggest that high levels of ethanol production by *C*. *phytofermentans* may be due to a combination of factors. These include: 1) increasing the energetic yield of glycolysis by utilizing pyrophosphate-dependent enzymes; 2) high levels of expression of the enzymes involved in ethanol production coupled with the ability to utilize both NADH and NADPH for ethanol biosynthesis; 3) the presence of multiple pathways for the dissipation of excess reducing equivalents; and 4) the presence of sodium-dependent energy generating pathways. Experimental studies will be required to determine if these hypotheses are indeed valid. Examination of the genomes of several well-studied cellulolytic and solventogenic clostridia indicated that very few of the central metabolic enzymes and complexes discussed above are unique to *C*. *phytofermentans*. It may therefore be the specific combination of enzymes and their transcriptional regulation that makes *C*. *phytofermentans* metabolism unique.

Efficient direct conversion of biomass to bioproducts using a microbial catalyst such as *C*. *phytofermentans* requires an increased understanding of cell growth dynamics, rate-limiting steps of biomass conversion, enzyme production and regulation. These genome-based experiments and analyses provide a blueprint for identifying bottlenecks and guiding strategies to generate novel productive strains for specific uses.

## Supporting Information

S1 File Phages in *C*. *phytofermentans*. (PDF)

S2 File Genes involved in sporulation. (PDF)

S3 File Bacterial microcompartment (BMC) loci genes. (PDF)

S4 File Genes involved in complex carbohydrate metabolism: diversity, functions, and origins. (PDF)

S5 File Expression data from microarray: raw expression values, averages and standard deviations, percentile ranking, ratio compared to glucose. (XLS)

S6 File Pentose metabolism. (PDF)

S7 File Investigation of *C*. *phytofermentans* central metabolism: Insights into high levels of ethanol production. (PDF)
