# Supplementary material for: Genome and Transcriptome of Clostridium phytofermentans, Catalyst for the Direct Conversion of Plant Feedstocks to Fuels
Source: PLoS One. 2015 Jun 2;10(6):e0118285. doi: 10.1371/journal.pone.0118285 (PMC4452783; doi:10.1371/journal.pone.0118285)
Supplement: S1 File — (PDF) [file pone.0118285.s001.pdf]

S1 File. Phages in *C. phytofermentans*

In order to determine whether the genome of *C. phytofermentans* contained evidence of phage infection, the sequences of all viruses (including phages) sequenced as of October 10, 2011, were downloaded from NCBI (ftp://ftp.ncbi.nih.gov/genomes/Viruses/) and used to search the predicted ORFs of *C. phytofermentans* for phage proteins using BLASTP. Two putative prophage-encoding clusters were identified, Cphy_0782-0798 and Cphy_2944-2997 (described in Table A). Both clusters contain genes for phage head and tail structural components, the majority of which, are homologous to those of *Clostridium difficile* phage ΦC2 (Goh et al. 2007). We were unable to identify homologs of many phage genes required for the completion of the phage lifecycle (genes involved in DNA packaging, tail assembly, cell lysis, and lysogeny control as well as DNA replication, recombination, and modification). This result suggests that these phages may no longer actively infect *C. phytofermentans.* Examination of the microarray data revealed differential transcription of the Cphy_0782-0798 cluster. It was upregulated during growth on cellulose, pectin and *Brachypodium distachyon* (relative to growth on glucose). In summary, the genome of *C. phytofermentans* encodes two clusters enriched in phage proteins, but it is not known whether these encoded phages are still active or affect the growth of the organism.

Table A. Prophage-like genes of *C. phytofermentans*.

Protein ID	Annotation	Homolog (Phage, e-value) [a]
**Cphy_0782**	XRE family transcriptional regulator	putative S24-like peptidase (*Geobacillus* virus E2, 1e-14)
**Cphy_0783**	hypothetical protein Cphy_0783	Gp2.21 (*Bacillus* phage SPO1, 0.019)
**Cphy_0784**	hypothetical protein Cphy_0784	hypothetical protein phiC2p12 (*Clostridium* phage phiC2, 3e-06)
**Cphy_0785**	phage protein	putative sheath tail protein (*Clostridium* phage phiC2, 4e-118)
**Cphy_0786**	phage protein	putative core tail (*Clostridium* phage phiC2, 7e-42)
**Cphy_0787**	XkdN-like protein	XkdN-like protein (*Clostridium* phage phiC2, 3e-21)
**Cphy_0788**	phage tape measure protein	putative tail tape measure protein (*Clostridium* phage phiC2, 7e-79)
**Cphy_0789**	peptidoglycan-binding LysM	putative LysM (*Clostridium* phage phiC2, 2e-27)
**Cphy_0790**	putative phage cell wall hydrolase	putative hydrolase (*Clostridium* phage phiC2, 1e-66)
**Cphy_0791**	hypothetical protein Cphy_0791	hypothetical protein phiC2p23 (*Clostridium* phage phiC2, 3e-09)
**Cphy_0792**	phage protein	hypothetical protein EJ-1p60 (*Streptococcus* phage EJ-1, 7e-19)
**Cphy_0793**	putative phage tail protein	hypothetical protein phiC2p25 (*Clostridium* phage phiC2, 1e-89)
**Cphy_0794**	hypothetical protein Cphy_0794	hypothetical protein phiC2p26 (*Clostridium* phage phiC2, 6e-18)
**Cphy_0795**	hypothetical protein Cphy_0795	hypothetical protein BC2598 (*Bacillus* phage phBC6A52, 0.086)
**Cphy_0796**	hypothetical protein Cphy_0796	nd
**Cphy_0797**	hypothetical protein Cphy_0797	nd
**Cphy_0798**	toxin secretion/phage lysis holin	Putative lysis protein (*Bacillus* virus 1, 9e-11)
**Cphy_2944**	hypothetical protein Cphy_2944	NLP/P60 family protein, enterotoxin (*Clostridium perfringens* SM101 phage, 6e-07)
**Cphy_2945**	toxin secretion/phage lysis holin	holin (*Bacillus* phage Fah, 9e-24)

Protein ID	Annotation	Homolog (Phage, e-value) [a]
**Cphy_2946**	hypothetical protein Cphy_2946	nd
**Cphy_2947**	hypothetical protein Cphy_2947	nd
**Cphy_2948**	hypothetical protein Cphy_2948	gp4 (*Mycobacterium* phage Bxz2, 0.012)
**Cphy_2949**	hypothetical protein Cphy_2949	hypothetical protein phiC2p26 (*Clostridium* phage phiC2, 4e-18)
**Cphy_2950**	baseplate J family protein	hypothetical protein phiC2p25 (*Clostridium* phage phiC2, 5e-90)
**Cphy_2951**	phage protein	hypothetical protein EJ-1p60 (*Streptococcus* phage EJ-1, 1e-17)
**Cphy_2952**	hypothetical protein Cphy_2952	hypothetical protein phiC2p23 (*Clostridium* phage phiC2, 1e-13)
**Cphy_2953**	putative phage cell wall hydrolase	putative hydrolase (*Clostridium* phage phiC2, 8e-61)
**Cphy_2954**	peptidoglycan-binding LysM	putative LysM (*Clostridium* phage phiC2, 2e-31)
**Cphy_2955**	hypothetical protein Cphy_2955	putative tail tape measure protein (*Clostridium* phage phiC2, 8e-43)
**Cphy_2956**	XkdN-like protein	hypothetical protein phiC2p16 (*Clostridium* phage phiC2, 1e-24)
**Cphy_2957**	phage protein	putative core tail (*Clostridium* phage phiC2, 3e-56)
**Cphy_2958**	phage protein	putative sheath tail protein (*Clostridium* phage phiC2, 1E-143)
**Cphy_2959**	hypothetical protein Cphy_2959	nd
**Cphy_2960**	phage protein	hypothetical protein phiC2p12 (*Clostridium* phage phiC2, 5e-28)
**Cphy_2961**	phage protein	hypothetical protein phiC2p11 (*Clostridium* phage phiC2, 3e-32)
**Cphy_2962**	phage protein	hypothetical protein phiC2p10 (*Clostridium* phage phiC2, 1e-26)
**Cphy_2963**	hypothetical protein Cphy_2963	hypothetical protein LJ771_034 (*Lactobacillus johnsonii* prophage Lj771, 8e-04)
**Cphy_2964**	phage coat protein	coat protein (*Enterococcus* phage phiFL3A, 1e-90)
**Cphy_2965**	hypothetical protein Cphy_2965	scaffold protein (*Enterococcus* phage phiFL3A, 4e-21)
**Cphy_2966**	hypothetical protein Cphy_2966	hypothetical protein lb338_phage_74 (*Lactobacillus* phage Lb338-1 ,7e-24)

Protein ID	Annotation	Homolog (Phage, e-value) [a]
**Cphy_2967**	hypothetical protein Cphy_2967	nd
**Cphy_2968**	hypothetical protein Cphy_2968	hypothetical protein phiC2p05 (*Clostridium* phage phiC2, 8e-06)
**Cphy_2969**	SPP1 family phage head morphogenesis protein	putative head morphogenesis protein (*Clostridium* phage phiC2, 3e-132)
**Cphy_2970**	SPP1 family phage portal protein	putative portal protein (*Clostridium* phage phiC2, 1e-103)
**Cphy_2971**	phage uncharacterized protein	TerL (*Lactococcus* phage TP901-1, 9e-151)
**Cphy_2972**	hypothetical protein Cphy_2972	hypothetical protein Tuc2009_28 (*Lactococcus* phage Tuc2009, 2e-43)
**Cphy_2973**	hypothetical protein Cphy_2973	nd
**Cphy_2974**	hypothetical protein Cphy_2974	hypothetical protein CST121 (*Clostridium* phage c-st, 5e-05)
**Cphy_2975**	hypothetical protein Cphy_2975	nd
**Cphy_2976**	hypothetical protein Cphy_2976	nd
**Cphy_2977**	hypothetical protein Cphy_2977	77ORF009 (*Staphylococcus* phage 77, 3e-10)
**Cphy_2978**	hypothetical protein Cphy_2978	
**Cphy_2979**	transcriptional regulator, CdaR	putative bacteriophage regulatory protein; Lambda gpCro analog (Phage Gifsy-2, 0.067)
**Cphy_2980**	hypothetical protein Cphy_2980	polyprotein precursor (GB virus A ,0.099)
**Cphy_2981**	hypothetical protein Cphy_2981	nd
**Cphy_2982**	hypothetical protein Cphy_2982	nd
**Cphy_2983**	hypothetical protein Cphy_2983	nd
**Cphy_2984**	superfamily II helicase	ORF001 (*Staphylococcus* phage PT1028, 3e-17)
**Cphy_2985**	zinc finger CHC2-family protein	primase (Abalone shriveling syndrome-associated virus, 0.008)
**Cphy_2986**	hypothetical protein Cphy_2986	nd
**Cphy_2987**	single-strand binding protein	SSB (*Lactococcus* phage bIL286, 6e-25)

Protein ID	Annotation	Homolog (Phage, e-value) [a]
**Cphy_2988**	hypothetical protein Cphy_2988	gp70 (*Mycobacterium* phage 244, 7e-30)
**Cphy_2989**	hypothetical protein Cphy_2989	hypothetical protein phi105_44 (*Bacillus* phage phi105, 2e-04)
**Cphy_2990**	hypothetical protein Cphy_2990	gp53 (*Listeria* phage P35, 1e-39)
**Cphy_2991**	metal dependent phosphohydrolase	nd
**Cphy_2992**	AbrB family transcriptional regulator	Transcription state regulatory protein abrB (*Bacillus* phage phBC6A51, 4e-14)
**Cphy_2993**	putative phage-related DNA binding protein	ORF032 (*Staphylococcus* phage X2, 4e-10)
**Cphy_2994**	XRE family transcriptional regulator	putative S24-like peptidase (*Geobacillus* virus E2, 1e-12)
**Cphy_2995**	hypothetical protein Cphy_2995	hypothetical protein phiETA3_gp05 (*Staphylococcus* phage phiETA3, 0.004)
**Cphy_2996**	hypothetical protein Cphy_2996	nd
**Cphy_2997**	resolvase domain-containing protein	putative phage site-specific recombinase (*Clostridium* phage phiCD27, 2e-78)

[a] *nd indicates not detected.*

References:

Goh S, Ong P, Song K, Riley T, Chang B. 2007. The complete genome sequence of *Clostridium difficile* phage phiC2 and comparisons to phiCD119 and inducible prophages of CD630. Microbiology (Reading, England). 153:676–685.
