# Supplementary material for: Genome and Transcriptome of Clostridium phytofermentans, Catalyst for the Direct Conversion of Plant Feedstocks to Fuels
Source: PLoS One. 2015 Jun 2;10(6):e0118285. doi: 10.1371/journal.pone.0118285 (PMC4452783; doi:10.1371/journal.pone.0118285)
Supplement: S2 File — (PDF) [file pone.0118285.s002.pdf]

S2 File. Genes involved in sporulation.

Sporulation. *C. phytofermentans,* like other members of the genus Clostridium, can form endospores (Cato et al. 1986). In previous studies, bioinformatic identification of sporulation-related genes has largely been based on sequence homology to those of *Bacillus subtilis* – the model organism for studying the sporulation cycle (Errington 2003; Hilbert & Piggot 2004; Barák & Wilkinson 2005). A comparative genomic review comparing the sporulation genes of *B. subtilis* and five clostridial species (*Clostridium acetobutylicum, Clostridium perfringens, Clostridium difficile, Clostridium botulinum, Clostridium tetani)* indicated that although the majority of the genes in the sporulation cascade of *B. subtilis* downstream of the master regulator SpoOA are present in Clostridial species, the ones upstream (the sensory histidine kinase and phosphorelay system) are not and the divergence in the first part of the sporulation cascade of the Bacilli and Clostridia may reflect the fact that they diverged from a common ancestor at very different times (after and before the Great Oxidation event, respectively) (Paredes et al. 2005). This also appears to be the case for *C. phytofermentans* (Table A described the genes found in the genome of *C. phytofermentans* and with a predicted function in sporulation).

The genome of *C. phytofermentans* contains a homolog of the master regulator of sporulation of *B. subtilis,* SpoOA (Cphy_2497, 55% amino acid identity to SpoOA of *B. subtilis*, Table A). (In the remainder of this section, all percentages will refer to amino acid identity with *B. subtilis* homologs.) A variety of other putative sporulation genes includes homologs of key sigma factors that are involved orchestrating the sporulation cascade of *B. subtilis*, *sigE*, *sigF* and *sigG* are present (Cphy_2469, Cphy_0478, and

Cphy_2468, 65%, 53% and 66%, respectively, Table A). The genome also contains several stage II sporulation genes (Table A) that are typically controlled by SpoOA in the prespore and are involved in asymmetric cell division are present in the genome (Paredes et al. 2005). The prespore-specific stage II genes include three putative operons: (1) the *spoIIA* operon consisting of the anti-anti-sigma factor *spoIIAA* (Cphy_0476, 36%), the anti-sigma factor *spoIIAB* (Cphy_0477, 53%), and the early forespore-specific sigma factor, *sigF* (Cphy_0478, 53%); (2) the *spoIIG* operon, consisting of *spoIIGA* (Cphy_2470, 22%) and *sigG* (Cphy_2468, 66%); (3) and Cphy_0135-0138, which contained a *spoIIE* homolog (Cphy_0138, 29%). Homologs of genes controlled by SpoOA in the mother cell are also present, and include the mother-cell-specific sigma factor, *sigE* (Cphy_2469, Table A). Homologs of genes involved in the later stages of sporulation, including stage III (engulfment of the prespore by the mother cell), stage IV (cortex formation), and stage V (spore coat formation) (Paredes et al. 2005) are also present (Table A).

Table A. Genes predicted to be involved in sporulation.

Protein ID	Predicted function	*Bacillus subtilis* homolog (% identity) [a]
Cphy_0050	sporulation protein YyaC	hypothetical protein BSU40950 (46)
Cphy_0051	peptidoglycan-binding LysM	na
Cphy_0109	stage V SpoVG family protein	regulatory protein SpoVG (65)
Cphy_0135	sporulation protein YabP	spore protein involved in the shaping of the spore coat (40)
Cphy_0136	spore cortex protein YabQ	na
Cphy_0137	septum formation initiator	na
Cphy_0138	stage II sporulation protein E (SpoIIE)	SpoIIE serine phosphatase (29)
Cphy_0210	stage III sporulation transcriptional regulator (SpoIIID)	SpoIIID transcriptional regulator (56)
Cphy_0476	stage II anti-anti-sigma factor (SpoIIAA)	SpoIIAA anti-anti-sigma factor (36)
Cphy_0477	stage II anti-sigma factor (SpoIIAB)	SpoIIAB anti-sigma F factor (53)
Cphy_0478	SigF early forespore-specific gene	SigF sporulation sigma factor (53)
Cphy_0479	stage V sporulation protein AC	spoVAC stage V sporulation protein AC (44)
Cphy_0480	stage V sporulation protein AD	SpoVAD stage V sporulation protein AD (44)
Cphy_0481	stage V sporulation protein AE	SpoVAEB spore germinant protein (43)
Cphy_0827	spore germination protein KB	GerKB spore germination receptor subunit (21)
Cphy_0828	spore germination protein KC	GerAC germination receptor GerA (23)
Cphy_0829	spore germination protein KA	GerKA spore germination receptor subunit (37)
Cphy_2316	stage II sporulation P family protein	SpoIIP spore autolysin (29)
Cphy_2317	GPR endopeptidase	Gpr germination protease (40)
Cphy_2385	stage IV sporulation protein A	SpoIVA morphogenetic stage IV sporulation protein (42)
Cphy_2468	SigG	SigG sporulation sigma factor SigG (66)
Cphy_2469	SigE mother-cell-specific sigma factor	SigE sporulation sigma factor SigE (65)
Cphy_2470	stage II sporulation protein (SpoIIGA)	SpoIIGA protease processing pro-sigma-E (22)
Cphy_2496	glycogen/starch synthase, ADP-glucose type	GlgA glycogen synthase (42)
Cphy_2497	sporulation transcriptional activator Spo0A	Spo0A response regulator (55)
Cphy_2498	stage IV sporulation protein B	SpoIVB regulatory membrane-associated serine protease (38)
Cphy_2517	stage III sporulation protein AH	na
Cphy_2518	stage III sporulation protein AG	spoIIIAG stage III sporulation engulfment assembly protein (31)
Cphy_2519	stage III sporulation protein AF	SpoIIIAF stage III Sporulation protein (23)
Cphy_2520	stage III sporulation protein AE	SpoIIIAE stage III sporulation protein (22)
Cphy_2521	stage III sporulation protein AD	SpoIIIAD stage III sporulation protein (39)
Cphy_2522	stage III sporulation protein AC	na
Cphy_2523	stage III sporulation protein AB	SpoIIIAB stage III sporulation protein SpoAB (27)
Cphy_2524	stage III sporulation protein AA	SpoIIIAA ATP-binding stage III sporulation protein (44)

Protein ID	Predicted function	*Bacillus subtilis* homolog (% identity) [a]
Cphy_2611	PhoH family protein	PhoH phosphate starvation-induced protein (55)
Cphy_2612	putative stage IV sporulation YqfD	YqfD stage IV sporulation protein (25)
Cphy_2613	protein of unknown function DUF1429	YqfC hypothetical protein (33)
Cphy_2726	sporulation protein YlmC/YmxH family	YlmC hypothetical protein (40)
Cphy_3479	stage II SpoIID/LytB domain protein	SpoIID stage II sporulation autolysin (34)
Cphy_3792	stage II sporulation protein R SpoIIR	SpoIIR pro-sigma E endopeptidase (34)
Cphy_3793	4-diphosphocytidyl-2C-methyl-D-erythritol kinase	Ipk 4-diphosphocytidyl-2-C-methyl-D-erythritol kinase (45)
Cphy_3794	peptidoglycan-binding LysM	LytF gamma-D-glutamate-meso-diaminopimelate muropeptidase (34)
Cphy_3824	spore coat protein, CotS family	CotI spore coat protein (20)
Cphy_3825	hypothetical protein	na
Cphy_3826	hypothetical protein	na
Cphy_3838	sporulation integral membrane protein YtvI	YtvI permease (27)
Cphy_3907	nucleoside recognition domain protein	SpmB spore maturation protein (37)
Cphy_3908	spore maturation protein	SpmA spore maturation protein (42)

[a] *na indicates not available.*

References:

Barák I, Wilkinson AJ. 2005. Where asymmetry in gene expression originates. Mol. Microbiol. 57:611-620.

Cato EP, George WL, Finegold S. 1986. Genus Clostridium. In: Bergey's Manual of Systematic Bacteriology.Vol. 2 P.H.A. Sneath, N.S. Mair, M.E. Sharpe, J.G. Holt: Baltimore, MD, USA pp. 1141–1200.

Errington J. 2003. Regulation of endospore formation in *Bacillus subtilis*. Nat. Rev. Microbiol. 1:117-126.

Hilbert D, Piggot P. 2004. Compartmentalization of gene expression during *Bacillus subtilis* spore formation. Microbiol. Mol. Biol. Rev. 68:234-262.

Paredes C, Alsaker K, Papoutsakis E. 2005. A comparative genomic view of clostridial sporulation and physiology. Nat. Rev. Microbiol. 3:969-978.

Warnick T, Methé B, Leschine S. 2002. *Clostridium phytofermentans* sp. nov., a cellulolytic mesophile from forest soil. Int. J. Syst. Evol. Microbiol. 52:1155-1160.
