# Supplementary material for: Genome and Transcriptome of Clostridium phytofermentans, Catalyst for the Direct Conversion of Plant Feedstocks to Fuels
Source: PLoS One. 2015 Jun 2;10(6):e0118285. doi: 10.1371/journal.pone.0118285 (PMC4452783; doi:10.1371/journal.pone.0118285)
Supplement: S3 File — (PDF) [file pone.0118285.s003.pdf]

S3 File. Bacterial microcompartment (BMC) loci genes

Protein ID	Best match in the *pdu* locus of *Salmonella enterica*	Best match in the *eut* locus of *Salmonella enterica*	Predicted function	Top Pfam
**Locus 1**				
**Cphy1174**	-	-	propanediol dehydratase	pfam02901
**Cphy1175**	-	-	propanediol dehydratase activating enzyme	pfam04055
**Cphy1176**	*pduU*	*eutS*	conserved microcompartment protein	pfam00936
**Cphy1177**	-	-	putative aldolase	pfam00596
**Cphy1178**	*pduP*	*eutE*	CoA-dependent propionaldehyde dehydrogenase	pfam00171
**Cphy1179**	-	-	propanol dehydrogenase	pfam00107
**Cphy1180**	*pduA*	*eutM*	Shell protein	pfam00936
**Cphy1181**	*pduJ*	*eutK*	Shell protein	pfam00936
**Cphy1182**	*pduA*	*eutM*	Shell protein	pfam00936
**Cphy1183**	*pduL*	-	phosphotransacylase	pfam06130
**Cphy1184**	*pduN*	*eutN*	CcmL polyhedral body protein	pfam03319
**Cphy1185**	*pduS*	-	putative cobalamin reductase	pfam01512
**Cphy1186**	*pduT*	*eutK*	Shell protein	pfam00936
**Locus 2**				
**Cphy1411**	*pduJ*	*eutM*	Shell protein	pfam00936
**Cphy1412**	-	-	possible choline transporter	pfam13536
**Cphy1413**	-	-	unknown function	NoMatch
**Cphy1414**	*pduJ*	*eutM*	Shell protein	pfam00936
**Cphy1415**	*pduJ*	*eutM*	Shell protein	pfam00936
**Cphy1416**	*pduP*	*eutE*	aldehyde dehydrogenase family	pfam00171
**Cphy1417**	-	-	possible propandiol dehydratase	pfam02901
**Cphy1418**	-	-	possible propandiol dehydratase activating enzyme	pfam04055

Protein ID	Best match in the *pdu* locus of *Salmonella enterica*	Best match in the *eut* locus of *Salmonella enterica*	Predicted function	Top Pfam
**Cphy1419**	*pduU*	*eutS*	conserved microcompartment protein	pfam00936
**Cphy1420**	*pduV*	*eutP*	*pdu* operon protein of unknown function - possible aspartokinase III	pfam10662
**Cphy1421**	*pduQ*	*eutG*	alcohol dehydrogenase	pfam00465
**Cphy1422**	-	-	weak similarity to cobalamin adenosyltransferase (EutT)	NoMatch
**Cphy1423**	-	*eutJ*	ethanolamine utilization protein	pfam11104
**Cphy1424**	*pduK*	-	Shell protein	pfam00936
**Cphy1425**	*pduN*	*eutN*	CcmL polyhedral body protein	pfam03319
**Cphy1426**	-	*eutQ*	putative ethanolamine utilization protein	pfam06249
**Cphy1427**	*pduJ*	*eutM*	Shell protein	pfam00936
**Cphy1428**	*pduP*	*eutE*	acetaldehyde dehydrogenase	pfam00171
**Cphy1429**	*pduL*	-	phosphotransacylase	pfam06130
**Cphy1430**	*pduJ*	*eutM*	Shell protein	pfam00936
**Locus 3**				
**Cphy2634**	-	*eutQ*	putative ethanolamine utilization protein	pfam06249
**Cphy2635**	-	*eutH*	ethanolamine transport protein	pfam04346
**Cphy2636**	*pduT*	*eutK*	Shell protein	pfam00936
**Cphy2637**	*pduN*	*eutN*	CcmL polyhedral body protein	pfam03319
**Cphy2638**	-	-	unknown function	NoMatch
**Cphy2639**	*pduL*	-	phosphotransacylase	pfam06130
**Cphy2640**	-	*eutT*	cobalamin adenosyltransferase	pfam01923
**Cphy2641**	*pduJ*	*eutM*	Shell protein	pfam00936
**Cphy2642**	*pduP*	*eutE*	aldehyde dehydrogenase	pfam00171

Protein ID	Best match in the *pdu* locus of *Salmonella enterica*	Best match in the *eut* locus of *Salmonella enterica*	Predicted function	Top Pfam
**Cphy2643**	*pduA*	*eutM*	Shell protein	pfam00936
**Cphy2644**	*pduB*	*eutL*	putative carboxysome structural protein	pfam00936
**Cphy2645**	-	*eutC*	ethanolamine ammonia-lyase small subunit	pfam05985
**Cphy2646**	-	*eutB*	ethanolamine ammonia-lyase heavy chain	pfam06751
**Cphy2647**	-	*eutA*	reactivating factor for ethanolamine ammonia lyase	pfam06277
**Cphy2648**	*pduV*	*eutP*	pdu operon protein of unknown function	pfam10662
**Cphy2649**	*pduU*	*eutS*	conserved microcompartment protein	pfam00936
**Cphy2650**	*pduQ*	*eutG*	ethanol dehydrogenase	pfam00465
