# Supplementary material for: Genome and Transcriptome of Clostridium phytofermentans, Catalyst for the Direct Conversion of Plant Feedstocks to Fuels
Source: PLoS One. 2015 Jun 2;10(6):e0118285. doi: 10.1371/journal.pone.0118285 (PMC4452783; doi:10.1371/journal.pone.0118285)
Supplement: S4 File — (PDF) [file pone.0118285.s004.pdf]

S4 File. Genes involved in complex carbohydrate metabolism: diversity, functions, and origins.

C. phytofermentans is among the few cultured anaerobic microbes capable of direct breakdown and fermentation of recalcitrant, insoluble lignocellulose components of plant cell walls. Lignocellulose consists of cellulose fibrils that are embedded in a complex matrix of hemicellulose, pectin and lignin. Lignocellulose breakdown requires the orchestration of diverse carbohydrate-active (CAZy) enzymes and associated proteins (Leschine 2005; Davies & Henrissat 2002). As detailed below, comparative genomic, transcriptional, and phylogenetic analyses were performed to better understand the mechanism and the evolution of lignocellulose degradation by *C. phytofermentans*. An up-to-date classification of enzymes that degrade, modify, or create glycosidic bonds is maintained in the CAZy website (www.cazy.org).

Diversity of CAZy enzymes

We began our inquiry into the ability of *C. phytofermentans* to degrade lignocellulose by quantifying the number and functional diversity of the CAZy domains encoded in the genome. The *C. phytofermentans* genome contains a large number and diversity of genes encoding CAZy enzymes (Table A and Figure 3), including glycoside hydrolases (GH), polysaccharide lyases (PL) and carbohydrate esterases (CE). Notably, *C. phytofermentans* has a total of 116 GH domains with a wide variety of predicted functions distributed among 44 enzyme families (Table A and Figure 3) including but not limited to endo- and exo-cellulases, hemicellulases, chitinases, pectinases, amylases, and

lichenases. The presence of such a large number of GHs with such a vast array of functions in a single genome is remarkable. Only the GH content of a distant relative in Cluster I, *Clostridium cellulovorans*, is comparable, with 113 GH domains distributed among 37 families (Figure 3). A closer relative of *C. phytofermentans*, *Butyrivibrio proteoclasticus* (Figure 3), has a comparable number of GH domains (113), but less diversity with only 25 families and no exo-cellulase (GH48). *Ca. saccharolyticus* is an interesting comparison as it is able to degrade switchgrass (Talluri et al. 2013)but has less GH domains than *C. phytofermentans*.

Comparative genomic and transcriptional analysis of CAZy enzymes

We conducted genomic and global gene expression analyses to identify which enzymes are likely involved in the degradation of specific components of lignocellulose. Gene expression was measured during growth on both complex lignocellulosic substrates ("plant feedstock"-- *Brachypodium distachyon*) and individual components of lignocellulose (xylan, cellulose, pectin and simple pentoses and hexoses) using genome-wide microarrays. By comparing gene expression on these substrates to gene expression on glucose, we identified enzymes that appear to play a significant role in growth of *C. phytofermentans* on lignocellulose.

Cellulose degradation. Current understanding of the hydrolysis of cellulose is limited. Cellulases (beta-1,4-glucanases) hydrolyze beta-1,4-glucosidic bonds (Leschine 2005). Endo-cellulases (EC 3.2.1.4) of the families GH5 and GH9 break internal bonds and may disrupt the crystalline structure of cellulose exposing individual cellulose polysaccharide chains (Leschine 2005). Exo-cellulases (or cellobiohydrolases) (EC 3.2.1.91), most

generally of the family GH48, cleave the ends of the exposed chains produced by endo-cellulases. However, GH5, GH9 and GH48 families have sometimes been reported to function as both exo- as well as endo-glucanases. The *C. phytofermentans* genome contains three putative GH5 endo-cellulases (Cphy_1163, 2058, 3202), a single GH9 endo-cellulase (Cphy_3367), and a single GH48 exocellulase (Cphy_3368) (Table A). Only three of these cellulases (GH9 Cphy_3367, GH48 Cphy_3368, and GH5 Cphy_1163) were highly expressed during growth on cellulose relative to glucose (Table B). The crucial role of the GH9 endo-cellulase for growth of *C. phytofermentans* on cellulose was confirmed by genetic analysis (Tolonen et al. 2009). A GH9-deficient strain was unable to degrade cellulose. Unexpectedly, four GHs with predicted functions unrelated to the hydrolysis of cellulose were also highly expressed on cellulose and a variety of lignocellulosic growth substrates. These included one beta-mannase (GH26 Cphy_1071), two chitinases (GH18 Cphy_1799, Cphy_1800), and one xylanase (GH11 Cphy_2105). The concurrent expression of these GHs on cellulose suggests that either these proteins have a cellulase activity, or that they may help *C. phytofermentans* to gain access to cellulose, which is rarely encountered in pure form in nature, by degrading other polysaccharides typically associated with cellulose. Alternatively, these proteins may be co-expressed members of the same regulon even though they have unrelated roles.

Hemicellulose degradation. In contrast to many other bacteria of biotechnological interest, *C. phytofermentans* has the ability to degrade and ferment both cellulose and hemicellulose, another plant cell wall cross-linking glycan. Hemicellulose is a major

component of plant biomass, where it accounts for 20-30% of the dry weight of woody tissue (Aspinall 1980). It is a structurally and chemically heterogeneous polymer, consisting primarily of a mixture of pentoses and hexoses such as D-xylopyranose, L-arabinofuranose, D-mannopyranose, D-glucopyranose, D-galactopyranose, D-glucopyranosyluronic acid and minor amounts of other sugars (Han & Rowell 1997). Hemicellulose is usually organized as a linear backbone consisting of one repeating sugar unit linked in beta-(1-4) with branch points, (1-2), (1-3) and/or (1-6). It also contains acetyl and methyl substituted groups. Therefore, enzymatic hydrolysis of hemicellulose requires an array of enzymes active on the backbone and side-chains, as well as on the different types of sugars. *C. phytofermentans* possesses an extensive library of domains putatively involved in hemicellulose degradation. The *C. phytofermentans* genome contains numerous domains that are predicted to be active on the linear backbone of hemicellulose: 16 endo-beta-1,4-D-xylanases (one GH8, six GH10, one GH11, eight GH43- EC 3.2.1.8), eight 1,4-beta-D-xyloside xylohydrolases (8 GH43- EC 3.2.1.37), and two beta-mannanases (two GH26, EC 3.2.1.78) domains. The genome of *C. phytofermentans* genome also encodes multiple domains predicted to be active on the side groups and constituents: 17 alpha-L-arabinofuranosidase (eight GH3, eight GH43, one GH51 -EC 3.2.1.55), one xylan α-1,2-glucuronidase (one GH67, EC 3.2.1.131), three alpha-glucosidase (three GH31 - EC 3.2.1.-), three alpha-fucosidase (three GH29, EC 3.2.1.51), 19 galactosidase (one GH1, five GH2, three GH4, two GH36, eight GH43- EC 3.2.1.23) and ten acetyl-xylan esterase (two CE2, seven CE4, one CE12 - EC 3.1.1.72) domains.

Only a subset of the putative hemicellulases were up-regulated on birchwood xylan and plant feedstocks relative to glucose: four xylanases (GH8 Cphy_3207, GH10 Cphy_2108, Cphy_3010 and GH11 Cphy_2105), one alpha-L-arabinofuranosidase (GH3 Cphy_3009) and one xylan α-1,2-glucuronidase (one GH67, EC 3.2.1.131) (Table C). In as much as hemicellulose composition varies tremendously with plant species and age, it is not surprising that only a few of the putative hemicellulases were up-regulated during growth on birchwood xylan (Table C). This result also suggests that *C. phytofermentans* can differentiate among the different hemicellulose components and specifically up-regulate the relevant enzymes.

Pectin degradation. Pectin is a naturally occurring polymer composed of galacturonic acid monomers. In plant cell walls, pectins cross-link cellulose and hemicellulose into a rigid structure and in certain plant types represent a significant portion, about one third, of the total cell-wall material (Rokas et al. 2007). Pectins are heterogeneous polysaccharides classified into four main types: homogalacturonan (HG), rhamnogalacturonan I (RG-I), rhamnogalacturonan II (RG-II), and xylogalacturonan (XGA). HG is a linear polymer of 1,4-alpha-linked D-galacturonic acid (D-galA) units. The carboxy group of the galacturonic acid residues can be methylated or acetylated (Ridley et al. 2001). RG is composed of alternating 1,2-alpha-L-rhamnose (L-rha) and D-galA residues (Lau et al. 1985). The rhamnose residues of the backbones usually have galactan, arabinan or arabinogalactan attached to C4 as side chains. The RG are highly modified on the L-rha and also partially acetylated at the C2 and C3 positions of D-galA. The main monosaccharides which can be liberated from pectin are D-galA, L-rha, D-

glucuronic acid, L-galactose, D-galactose, L-arabinose, D-xylose, and L-fucose.HGs are degraded by pectate lyases. These enzymes are predominantly endolytic and extracellular (Abbott & Boraston 2008). Pectate lyases typically contain domains within six families, PL1, PL2, PL3, PL9, PL10, and PL22 (Garron & Cygler 2010).

In this study, we cultured *C. phytofermentans* on apple pectin, which is a poly-galacturonic acid methyl ester**.** Genes encoding putative secreted pectate lyases containing PL domains were highly up-regulated compared to glucose. These included a multimodular PL1-PL9 enzyme (Cphy_1612) and three PL1 enzymes (Cphy_1888, Cphy_2919, Cphy_3869) (Table D).

Enzymes cleaving RG have been less studied. Rhamnogalacturonan lyases (RLs) cleave glycosidic bonds between L-rha and D-galA. RLs are classified in two families, PL4 and PL11 (Garron & Cygler 2010). The genome of *C. phytofermentans* harbors a gene (Cphy_0343), which encodes a putative RL (PL11) domain, domain, but it is only up-regulated 1.6 fold on apple pectin compared to glucose (Table D). It is more highly expressed on switchgrass, which might reflect its higher RG pectin content. Cphy_0343 is likely part of an operon (Cphy_0339-0343) encoding a putative regulator and a putative transporter (Table D), which may possibly function together as a unit. The most highly up-regulated GH during growth on apple pectin is Cphy_3586-GH53 (Table D). Cphy_3586 is a putative arabinogalactan endo-beta-1,4-galactanase (Ryttersgaard et al. 2004) and is encoded in a cluster (Cphy_3585-3590) containing a putative transporter and transcriptional regulator (Table D). The role of this enzyme would be to hydrolyze beta-

1,4- galactosidic linkages of the type I arabinogalactan side chains of RG, releasing D-galactose and oligogalactans (Ryttersgaard et al. 2004).

Hydrolysis of glycosidic bonds between L-rha and D-galA within pectic fragments is typically catalyzed by GH28 polygalacturonases (Abbott & Boraston 2008). *C. phytofermentans* has 5 putative polygalacturonases but only one, Cphy_3310, is slightly differentially expressed on apple pectin in comparison to glucose (data not shown).

Two operons Cphy_2263-2276, Cphy_1714-1723 are potentially involved in the metabolism of side chains of RG and encode a PH1107-related enzyme, transporters and transcriptional regulators (Table D). PH1107-related enzymes are glycosidases or glycosyl hydrolases, which based on sequence homology belong to the beta-fructosidase superfamily (Hunter et al. 2012) that hydrolyse the glycosidic bonds between carbohydrates or between a carbohydrate and an aglycone moiety and that most probably, act on a furanoside residue (fructose, arabinose and ribose) (Naumoff 2001). The Cphy_1714-1723 operon is differentially expressed on apple pectin (Table D). In addition to the PH1107-related enzyme, it encodes a putative multimodular (GH85-CBM32) endo-beta-N-acetylglucosamidase multimodular GH85-CBM32 enzyme. In *Yersinia enterocolitica*, CBM32 interacts with polygalacturonic acid, a central component of pectin (Abbott & Boraston 2008).

Five glucuronyl hydrolase domain containing proteins (four GH88 and one GH105, Table D) may cleave the oligosaccharide products of lyases with a 4,5-unsaturated residue at the non-reducing end. However, with the exception of Cphy_0288

(Table D), which seems to be part of an operon with transporters and regulators (Cphy_0288-0293), these genes were not up-regulated during growth on apple pectin. *C. phytofermentans* also has one putative pectin acetylesterase domain(CE12) to remove the acetyl groups and one putative pectin methylesterase domain(CE8) to remove the methoxyl groups, releasing methanol and converting methylgalacturonate residues to galacturonate residues, or pectin to pectate. However, neither of these enzymes was transcriptionally up-regulated during growth on apple pectin in comparison to glucose.

Lignocellulose degradation. When *C. phytofermentans* was grown on three different plant feedstocks, GHs expression profiles were similar to each other and to cellulose profiles, with the exception of higher levels of expression of one xylanase (Cphy_2105) and one mannanase (Cphy_1071) during growth on feedstock compared to cellulose (data not shown). This result suggests that analysis of differential gene expression on individual components of lignocellulose is a valid strategy for deciphering the functions of the multitude enzymes involved in lignocellulose degradation.

Starch, chitin and lichenin degradation. *C. phytofermentans* degrades not only complex plant cell wall material but a variety of other glycans including starch, chitin and lichenin (Leschine 2005; Reguera & Leschine 2001; Warnick et al. 2002). Starch molecules are cleaved to oligosaccharides by a diversity of alpha-amylases (GH13). The resulting oligosaccharides may be further hydrolyzed by glucoamylases, (beta-amylases and other exo-alpha-1,4-glucanases) and pullulanases which hydrolyze alpha-1, 6 branches. The genome of *C. phytofermentans* encodes 7 putative starch-degrading enzymes with a GH13 domain (Table A). Chitin is a long-chain polymer of a N-

acetylglucosamine, a derivative of glucose, and is found is the main component of the cell walls of fungi and the exoskeletons of arthropods and insects. The *C. phytofermentans* genome includes eight genes with potential chitinase activity (six GH18 and two GH19) (Table A). Curiously, two of these are up-regulated during growth on a variety of substrates including cellulose (Table B) and plant feedstocks (data not shown), suggesting either that *C. phytofermentans* often encounters chitin in its natural environment or that these genes have an alternate substrate specificity. Finally, lichenin is a complex glucan occurring in certain species of lichens. Chemically, lichenin consists of repeating glucose units linked by beta-1,3 and beta-1,4 glycosidic bonds. *C. phytofermentans* possesses one lichenase (GH16, Cphy_3388), which could assist in the decomposition of additional cell wall cross-linking glycans, the beta (1,3)-glucans and mixed linkage beta (1,3), (1,4)-glucans (Table A).

Plant cell wall degradation without a cellulosome

Absence of a cellulosome. Cellulosome complexes are believed to play an important role in plant cell wall breakdown by cellulolytic clostridia, such as *Clostridium cellulolyticum* and *Clostridium thermocellum,* by enabling the bacteria to concentrate and orient a diversity of enzymes involved in cleaving the various linkages in plant cell wall polysacchacharides at the cell surface. Cellulosomes may maximize the stoichiometry and the synergy between enzymes with different catalytic and binding specificities; and they may also limit the diffusion of breakdown products away from the cell by sequestering them between the cell membrane and the substrate (Flint et al. 2008). They are encoded by conserved genetic loci that include genes for cellulosome

integration proteins (Cip). Cip proteins contain type I cohesin domains that act as receptors for type I dockerin domains found in cellulosomal enzymes such as cellobiohydrolases, pectinases an endoglucanases. Surprisingly, there is no genomic evidence for the production of cellulosomes by *C. phytofermentans*. The *C. phytofermentans* genome does not contain genes homologous to cellulosome anchoring proteins or genes harboring cohesin and dockerin domains.

Multi-modular enzymes. Despite the absence of cellulosomal assembly domains, the striking multimodular nature of cellulosomal proteins, in which multiple domains from diverse families of GH, CE, PL and carbohydrate-binding modules (CBM) are found within individual proteins is preserved in *C. phytofermentans* (Table A). *C. phytofermentans* has 19 multimodular GH proteins, representing about 17% of all putative GH genes (Table A). In fact, the largest protein in the proteome is the multimodular glycoside hydrolase family 10 protein Cphy_3862, with 2457 amino acids and a predicted molecular weight of 266 kD (Tolonen et al. 2011). This protein contains consecutive GH10, CE15, and CBM domains. In non-cellulolytic bacteria, the corresponding GH domains are found mainly in single-domain polypeptides, which are cytosolic and act on smaller, soluble carbohydrate substrates (Flint et al. 2008). Thus, the multi-modular organization that seems to be characteristic of enzymes from cellulolytic species, may reflect their involvement in the extracellular processing of heterogeneous insoluble substrates, such as plant cell walls (Flint et al. 2008).

Carbohydrate binding modules. In the absence of a cellulosome, CBMs may enable the enzymes involved in lignocellulose degradation to adhere firmly to the plant cell wall

substrate, thereby facilitating the degradation of the heterogeneous topologies of highly cross-linked lignocellulose polysaccharides. Thirty-five putative CBMs representing 15 CAZy families were identified in the *C. phytofermentans* genome (Table A). CBM2, CBM3, CBM4, CBM6 and CBM46 have been shown to bind cellulose (Table A), whereas CBM2, CBM4, CBM6, CBM13, CBM22, CBM35, and CBM36 have been demonstrated to bind xylan (Table A). All but one of the CBM domains are found within multimodular proteins, the majority of which also contain catalytic domains (Table A). The genome contains multiple examples of multimodular GH in which catalytic domains are combined with CBM domains with specificities that differ from those of the catalytic domains. This may be advantageous for the degradation of plant cell wall topologies in which multiple polysaccharide types are cross-linked. For example, xylanases with cellulose-binding CBMs might help *C. phytofermentans* attach to cellulose fibers while degrading the cross-linked xylan. CBMs in proteins lacking catalytic domains may potentially play a thermostabilizing role (Charnock et al. 2000).

Another type of domain, alternatively named DUF291, hydrophilic domain (Pagès et al. 1999), or X2 module (Mosbah et al. 2000), is found in conjunction with CBMs and catalytic domains in a putative mannanase (Cphy_2128) and in three putative cellulases (Cphy_3202, Cphy_3367, Cphy_3368) (Table A). This type of domain is found in the cellulosomal scaffoldin proteins of *C. cellulolyticum* (CipC), *C. cellulovorans* (CbpA), and *C. josui* (CipA). The CbpA DUF291 domain binds both cellulose and the cell wall (Kosugi et al. 2004), suggesting that it promotes cellulose degradation by helping to anchor the cellulosome to the substrate and the cell surface.

Perhaps this type of domain plays a similar role in *C. phytofermentans,* enabling selected GHs to associate with their substrates and the cell surface simultaneously.

Cell surface-binding domains. In addition to binding to polysaccharides, many of the multimodular enzymes of *C. phytofermentans* appear to be cell bound. The majority of the 31 GH enzymes that are predicted to be secreted (Gardy et al. 2005) also have domains potentially involved in anchoring them to the either the membrane or the cell wall including, transmembrane helices, TonB boxes (COG0810 and PS00430, (e-value <1.00E-10)) and/or CBM domains. Among these proteins, 8 GH enzymes are predicted to have both CBM and cell attachment capability, and may play a role in keeping the *C. phytofermentans* in close proximity to its growth substrate. For example, the unusual gene Cphy_1775 (SLH-GH*-CBM32-CBM32) contains a catalytic domain, an SLH domain (pfam00395) for anchoring it to the cell wall and also two immunoglobulin-like folds (CBM32) that may behave like a CBM domain. Other GH enzymes might be anchored to the cell surface by as yet unknown mechanisms.

Biofilm formation may also play an important role in the orchestration of the degradation of the plant cell wall polysaccharides. Cells might adhere to each other via a variety of different domains such as pfam07705 (CARDB, cell adhesion domain in bacteria) and pfam01391 (Collagen, Collagen triple helix repeat), both of which are found in the *C. phytofermentans* genome.

Genomic analysis suggests that many GH enzymes are secreted and not cell-associated. In this case, what strategy might *C. phytofermentans* use to impede its competitors from utilizing the degraded products? It is possible that *C. phytofermentans*

does not degrade its substrates to monosaccharides or other simpler products that could be used by its competitors at the cell surface. Rather, *C. phytofermentans* might degrade the polysaccharides to more complex oligosaccharides that it can uptake and degrade to monosaccharides intracellularly. This hypothesis is supported by the high levels of expression of intracellular xylosidases (Cphy_3009, Cphy_3207) and cellodextrin phosphorylases (Cphy_3854, Cphy_0430, Cphy_1929) (Tolonen et al. 2011). Alternatively, freely secreted enzymes may remain close to the cell if they are embedded in a biofilm-related polysaccharide matrix.

In summary, *C. phytofermentans* lacks the structural elements of the cellulosome, but does have the functional equivalents of all cellulosome-associated hydrolytic enzymes and possesses many multimodular enzymes that also contain carbohydrate binding and/or cell surface binding domains. The simultaneous expression of combinations of these proteins may play a role similar to that of the cellulosome and may help to keep the lignocellulose-degrading enzymes of *C. phytofermentans* in close proximity to both the cell surface and their substrates. From a biotechnological standpoint, a cellulase system that is not cellulosome associated is very attractive. It is more flexible than a cellulosome in that individual enzymes can more easily be modified, introduced, or transferred to another organism.

Origins of GHs.

As described above, the number and diversity of the GHs encoded in the *C. phytofermentans* genome is remarkable. Below we present a possible explanation for the evolution of the plant degradative abilities of *C. phytofermentans* based on comparative

genomic analyses, investigation of the physical arrangement of the GH genes within the genome, and phylogenetic analysis of selected GHs.

Assembly of a unique set of glycoside hydrolases through horizontal gene transfer. To gain insight into the origin of the GHs of *C. phytofermentans*, we identified the closest relatives of the GHs of *C. phytofermentans* in the GenBank database using BLASTP and compared their distribution to that of the closest relatives of all of the protein-coding genes within the *C. phytofermentans* genome. The latter analysis was performed to calibrate how much similarity to other bacteria would be expected on average. Only 20% of closest relatives of the GH's of *C. phytofermentans* were within clostridial cluster XIVA (Figure 4) vs. 35% for all of the genes in the genome. Of the remaining GH genes, 20% were most similar to those of Bacilli, followed by 15% and 8% within clostridial clusters I and III respectively, both of which contain well studied cellulolytic organisms (Figure 4). In total, approximately 40% of the GHs of *C. phytofermentans* were most similar to GHs present in species outside the class Clostridia, whereas only 20% of all the genes in the *C. phytofermentans* genome were most similar to genes from outside the Clostridia. The higher than expected proportion of GHs with distant relatives is statistically significant (Pearson's Chi-squared test, X-squared= 77.8583, df = 9, p-value = 4.299e-13) (Figure 4). This result suggests that horizontal gene transfer from diverse origins rather than vertical divergence from an ancestral genome played a key role in the assembly of the unique set GHs present in *C. phytofermentans*. The association of enzymes from diverse unrelated bacteria in the genome of *C. phytofermentans* may

reflect the diversity of the community typically associated with *C. phytofermentans* in its natural environment (Hehemann et al. 2010).

Conservation of highly expressed lignocellulosic enzymes among the *Clostridia*. In order to investigate which of the crucial lignocellulosic enzymes that were identified via microarray analysis (Table B-8) were inherited from distantly related clostridia and which were present in close relatives, we carried out genome-wide comparison of all fully sequenced clostridial genomes. *Clostridium saccharolyticum* in cluster XIVa shares the most genes with *C. phytofermentans* (1861 BLASTP hits, e-value <10-20). However, *C. saccharolyticum* lacks most of the highly expressed lignocellulosic GHs described above. Within cluster XIVa, *Butyrivibrio proteoclasticus* has a comparable number of GHs (Figure 3) but overall its genome has fewer genes in common with *C. phytofermentans* (1480 BLASTP hits; e-value <10-20) than *C. cellulovorans*, a member of cluster I (1505 BLASTP hits; e-value<10-20). *B. proteoclasticus* has most of the highly expressed GHs of *C. phytofermentans*, with the exception of the exocellulase (GH48 Cphy_3368) and the two chitinases (GH18 Cphy_1799 and Cphy_1800). *C. cellulovorans* possesses all of the lignocellulosic enzymes that were highly expressed in *C. phytofermentans* with the exception of the chitinases. Thus, in terms of lignocellulose degradation, *C. cellulovorans* may be a better comparative model for *C. phytofermentans,* despite the fact that it belongs to a different taxonomical cluster within the Clostridia.

Genomic location of GHs. By exploring the physical arrangement of the genes in the genome, one can infer commonality of function and/or origin. The majority of GH genes are not co-localized with the exception of the starch-degradation genes (Cphy_2304-

2352) and the genes encoding the two main cellulases, GH9 (endo-cellulase, Cphy_3367) and GH48 (exocellulase, Cphy_3368). The fact that the majority of GHs are distributed throughout the genome rather than found in physical proximity is consistent with the hypothesis that the majority of the GHs of *C. phytofermentans* were acquired through horizontal gene transfer rather than gene duplication.

The starch-degradation genes seem to be part of an operon that has been transferred between many taxa. It is found in members of the Bacilli (some species in the genera *Bacillus*, *Paenibacillus*, *Geobacillus*) and in Clostridial clusters I (Collins et al. 1994) (in some strains of *C. botulinum,* and in *C. beijeirinckii* but not in *C. cellulovorans)* and X (Collins et al. 1994) (in the genus *Caldicellulosiruptor*).

The contiguity of the genes encoding the two main cellulases, GH9 (endo-cellulase) and GH48 (exocellulase) is consistent with the pair-wise interaction between these cellulases in characterized bacterial cellulase systems (Riedel et al. 1997) as well studies performed in *C. phytofermentans* (Tolonen et al. 2009; Zhang et al. 2010). The closest characterized relatives of *C. phytofermentans* GH9 and GH48 are found in the thermophilic cellulolytic and xylanolytic species, *Clostridium stercorarium:* the endoglucanase Z precursor (Avicelase I) (Jauris et al. 1990) and the cellodextrinohydrolase (Avicelase II), respectively (Bronnenmeier et al. 1991). In *C. stercorarium*, the GH9 and GH48 genes are also adjacent (Schwarz et al. 2004). In *Caldicellulosiruptor (Ca.) saccharolyticus* GH9 and GH48 are fused into a single protein. These observations suggest a common origin and pair-wise functioning of these key enzymes in these three bacteria.

Redundancy in CAZy functions. Generally speaking *C. phytofermentans* has a broad range of GH families with little redundancy within individual families, consistent with its nutritional versatility (Figure 3). This contrasts with the cellulose specialist *C. thermocellum*, which has large families of cellulase genes (16 GH9, 2 GH48, and 11 GH5) that enable it to rapidly degrade cellulose. Nevertheless several GH families in *C. phytofermentans* do contain numerous members (Table A). This is the case for the GH3 glucosidases and GH5 cellulases as well as GH10, GH26, and GH43 xylan-degrading enzymes. Among these, Cphy_2108 (GH10), which was highly expressed on birch xylan (Table C), is very similar to the multimodular xylanase of *C. stercorarium* Xyn10C, a thermostable, cell surface-associated, cellulose and xylan-binding protein (Adelsberger et al. 2004) . In another putative xylanase, Cphy_3862, two unique but closely related GH10 domains are located in tandem followed by a CE15 methylesterase domain) suggesting a duplication event followed by fusion and divergence. This specific gene is unique to *C. phytofermentans* and is expressed two to four fold higher on all plant feedstocks, cellulose, xylan and arabinose than on glucose.

Collectively, the similarity of GHs to remote relatives, the fact that they are broadly distributed throughout the genome, and the low amount of redundancy per gene family is consistent with the hypothesis that horizontal gene transfer rather than gene duplication, allowed the acquisition of a complex array of genes and gene clusters from other members of the ecological niche. Thus, this suggests that *C. phytofermentans* is in close contact with Bacilli (in the genera of *Geobacillus* and *Paenibacillus*) and Clostridia from cluster I (*C. beijeireinckii* and *C. cellulovorans*), III (*Clostridium cellulolyticum*)

and X (*Thermoanaerobacter thermosaccharolyticus* and species of *Caldicellulosiruptor*) in its natural environment.

Implications

Analyses of the *C. phytofermentans* genome revealed a large number and diversity of multimodular enzymes, that were likely to play a role in lignocellulose degradation and which appeared to be acquired primarily via horizontal gene transfer,. However, transcriptional profiling during growth of *C. phytofermentans* on plant feedstocks and a variety of individual lignocellulose components suggested that only a small subset of these genes were involved in growth on lignocellulose in the laboratory. Furthermore, experiments with individual lignocellulose components revealed that enzymes with predicted substrate specificities unrelated to the component present in the growth medium were often up-regulated. These discrepancies reveal that our ability to predict which genes are responsible for polysaccharide degradation based on their sequence is still limited and speak to the significance of expression arrays in deciphering the functions of these enzymes. They also point out the difficulties of trying to assess gene function during growth on simple substrates in an organism that is adapted for growth on complex substrates. Nevertheless, the analyses presented in this work are an important first step in the investigation of plant degradative properties of *C. phytofermentans* and its interactions with other organisms in its natural environment.

Table A. List of glycoside hydrolases (GHs), glycoside transferases(GTs), polysaccharide lyases (PLs), and carbohydrate-binding module (CBM)-containing proteins discovered in the *C. phytofermentans* genome (www.CAZy.org)

Protein ID	Predicted number	CAZy module(s)
Cphy_0110	Carbohydrate Esterase Family 4 protein	CE4
Cphy_0191	Glycoside Hydrolase Family 43 protein	GH43
Cphy_0203	Glycoside Hydrolase Family 105 protein	GH105
Cphy_0218	Glycoside Hydrolase Family 31 protein	GH31
Cphy_0220	Glycoside Hydrolase Family 3 protein	GH3
Cphy_0288	Glycoside Hydrolase Family 88 protein	GH88
Cphy_0297	Glycosyltransferase Family 2 protein	GT2
Cphy_0299	Glycosyltransferase Family 2 protein	GT2
Cphy_0323	Glycosyltransferase Family 2 protein	GT2
Cphy_0343	Polysaccharide Lyase Family 11 protein	PL11
Cphy_0405	Carbohydrate Esterase Family 4 protein	CE4
Cphy_0430	Glycoside Hydrolase Family 94 protein	GH94
Cphy_0488	Polysaccharide Lyase Family 15 protein	PL15
Cphy_0489	Polysaccharide Lyase Family 17 protein	PL17
Cphy_0492	Carbohydrate-Binding Module Family 50 / Polysaccharide Lyase Family 7 protein	CBM50-CBM50-PL7
Cphy_0497	Glycosyltransferase Family 51 protein	GT51
Cphy_0498	Glycosyltransferase Family 51 protein	GT51
Cphy_0530	Glycoside Hydrolase Family 2 protein	GH2
Cphy_0531	Glycoside Hydrolase Family 43 protein	GH43
Cphy_0538	Carbohydrate Esterase Family 4 protein	CE4
Cphy_0601	Carbohydrate Esterase Family 4 protein	CE4
Cphy_0607	Glycoside Hydrolase Family 20 protein	GH20
Cphy_0624	Carbohydrate-Binding Module Family 22 / Glycoside Hydrolase Family 10 protein	CBM22-GH10

Protein ID	Predicted number	CAZy module(s)
Cphy_0662	Glycoside Hydrolase Family 3 protein	GH3
Cphy_0694	Glycoside Hydrolase Family 94 protein	GH94
Cphy_0699	Glycoside Hydrolase Family 3 protein	GH3
Cphy_0711	Glycoside Hydrolase Family 2 protein	GH2
Cphy_0769	Glycoside Hydrolase Family 4 protein	GH4
Cphy_0776	Glycoside Hydrolase Family 88 protein	GH88
Cphy_0857	Glycoside Hydrolase Family 94 protein	GH94
Cphy_0858	Glycoside Hydrolase Family 30 protein	GH30
Cphy_0874	Glycoside Hydrolase Family 95 protein	GH95
Cphy_0875	Glycoside Hydrolase Family 43 protein	GH43
Cphy_0934	Glycoside Hydrolase Family 88 protein	GH88
Cphy_1019	Glycoside Hydrolase Family 65 protein	GH65
Cphy_1071	Carbohydrate-Binding Module Family 35 / Glycoside Hydrolase Family 26 protein	CBM35-GH26-CBM3
Cphy_1125	Glycoside Hydrolase Family 3 protein	GH3
Cphy_1140	Carbohydrate Esterase Family 2 protein	CE2
Cphy_1163	Glycoside Hydrolase Family 5 protein	GH5
Cphy_1169	Glycoside Hydrolase Family 51 protein	GH51
Cphy_1202 + Cphy_1203	Glycosyltransferase Family 1 protein [a]	GT1
Cphy_1204	Glycosyltransferase Family 4 protein	GT4
Cphy_1206	Glycosyltransferase Family 4 protein	GT4
Cphy_1254	Carbohydrate Esterase Family 2 protein	CE2
Cphy_1301	Carbohydrate Esterase Family 8 protein	CE8
Cphy_1308	Glycoside Hydrolase Family 87 protein	GH87
Cphy_1395	Glycoside Hydrolase Family 95 protein	GH95
Cphy_1435	Glycoside Hydrolase Family 19 protein	GH19
Cphy_1510	Glycoside Hydrolase Family 10 protein	GH10

Protein ID	Predicted number	CAZy module(s)
Cphy_1596	Glycoside Hydrolase Family 3 protein	GH3
Cphy_1612	Polysaccharide Lyase Family 1 / Polysaccharide Lyase Family 9 protein	PL1-PL9
Cphy_1614	Glycosyltransferase Family 51 protein	GT51
Cphy_1640	Glycoside Hydrolase Family 12 protein	GH12
Cphy_1645	Glycosyltransferase Family 2 protein	GT2
Cphy_1652	Carbohydrate-Binding Module Family 50 / Glycoside Hydrolase Family 18 protein	CBM50-CBM50-GH18
Cphy_1687	Carbohydrate-Binding Module Family 36 / Carbohydrate Esterase Family 4 protein	CBM36-CE4
Cphy_1695	Glycosyltransferase Family 2 protein	GT2
Cphy_1711	Glycoside Hydrolase Family 28 protein	GH28
Cphy_1713	Carbohydrate-Binding Module Family 32 protein	CBM32
Cphy_1714	Glycoside Hydrolase Family 85 / Carbohydrate-Binding Module Family 32 protein	GH85-CBM32
Cphy_1720	Glycoside Hydrolase Family 38 protein	GH38
Cphy_1750	Glycoside Hydrolase Family 105 protein	GH105
Cphy_1775	Non-Catalytic Module Family SLH / Carbohydrate-Binding Module Family 54 / Glycoside Hydrolase Family 55 / Carbohydrate-Binding Module Family 32 protein	SLH-SLH-CBM54-GH55-CBM32-CBM32
Cphy_1799	Carbohydrate-Binding Module Family 12 / Glycoside Hydrolase Family 18 protein	CBM12-GH18
Cphy_1800	Glycoside Hydrolase Family 18 / Carbohydrate-Binding Module Family 12 protein	GH18-CBM12
Cphy_1847	Carbohydrate Esterase Family 4 protein	CE4
Cphy_1873	Carbohydrate-Binding Module Family 35 / Carbohydrate-Binding Module Family 6 / Glycoside Hydrolase Family 87 protein	CBM35-CBM6-GH87
Cphy_1874	Glycoside Hydrolase Family 65 protein	GH65
Cphy_1877	Glycoside Hydrolase Family 31 protein	GH31
Cphy_1882	Glycoside Hydrolase Family 87 protein	GH87
Cphy_1888	Polysaccharide Lyase Family 9 protein	PL9
Cphy_1892	Glycosyltransferase Family 28 protein	GT28
Cphy_1919	Glycoside Hydrolase Family 105 protein	GH105
Cphy_1929	Glycoside Hydrolase Family 94 protein	GH94
Cphy_1934	Glycoside Hydrolase Family 13 protein	GH13

Protein ID	Predicted number	CAZy module(s)
Cphy_1936	Glycoside Hydrolase Family 36 protein	GH36
Cphy_1937	Glycoside Hydrolase Family 1 protein	GH1
Cphy_1943	Carbohydrate-Binding Module Family 5 / Glycoside Hydrolase Family 19 protein	CBM5-GH19
Cphy_2025	Glycoside Hydrolase Family 2 protein	GH2
Cphy_2028	Glycoside Hydrolase Family 43 protein	GH43
Cphy_2058	Glycoside Hydrolase Family 5 protein	GH5
Cphy_2105	Glycoside Hydrolase Family 11 protein	GH11
Cphy_2108	Carbohydrate-Binding Module Family 22 / Glycoside Hydrolase Family 10 protein	CBM22-GH10
Cphy_2128	Carbohydrate-Binding Module Family 35 / Glycoside Hydrolase Family 26 protein	CBM35-GH26-CBM3
Cphy_2190	Glycoside Hydrolase Family 29 protein	GH29
Cphy_2210	Glycoside Hydrolase Family 18 protein	GH18
Cphy_2212	Glycosyltransferase Family 2 protein	GT2
Cphy_2276	Carbohydrate-Binding Module Family 35 / Glycoside Hydrolase Family 26 protein	CBM35-GH26
Cphy_2304	Carbohydrate-Binding Module Family 41 / Carbohydrate-Binding Module Family 48 / Glycoside Hydrolase Family 13 protein	CBM41-CBM48-GH13
Cphy_2331	Carbohydrate-Binding Module Family 48 / Glycoside Hydrolase Family 13 protein	CBM48-GH13
Cphy_2332	Glycoside Hydrolase Family 3 protein	GH3
Cphy_2336	Glycosyltransferase Family 4 protein	GT4
Cphy_2341	Glycoside Hydrolase Family 13 protein	GH13
Cphy_2342	Glycoside Hydrolase Family 13 protein	GH13
Cphy_2344	Glycoside Hydrolase Family 13 protein	GH13
Cphy_2348	Glycosyltransferase Family 35 protein	GT35
Cphy_2349	Glycoside Hydrolase Family 77 protein	GH77
Cphy_2350	Glycoside Hydrolase Family 13 protein	GH13
Cphy_2496	Glycosyltransferase Family 5 protein	GT5
Cphy_2567	Glycoside Hydrolase Family 28 protein	GH28
Cphy_2572	Glycoside Hydrolase Family 18 protein	GH18

Protein ID	Predicted number	CAZy module(s)
Cphy_2632	Glycoside Hydrolase Family 43 protein	GH43
Cphy_2735	Carbohydrate Esterase Family 12 protein	CE12
Cphy_2736	Glycoside Hydrolase Family 28 protein	GH28
Cphy_2737	Glycoside Hydrolase Family 105 protein	GH105
Cphy_2807	Glycosyltransferase Family 2 protein	GT2
Cphy_2810	Glycosyltransferase Family 2 protein	GT2
Cphy_2811	Glycosyltransferase Family 4 protein	GT4-GT4
Cphy_2813	Glycosyltransferase Family 4 protein	GT4
Cphy_2814	Glycosyltransferase Family 2 protein	GT2
Cphy_2848	Glycoside Hydrolase Family 4 protein	GH4
Cphy_2887	Glycosyltransferase Family 2 protein	GT2
Cphy_2919	Polysaccharide Lyase Family 9 protein	PL9
Cphy_3009	Glycoside Hydrolase Family 3 protein	GH3
Cphy_3010	Glycoside Hydrolase Family 10 protein	GH10
Cphy_3011	Glycoside Hydrolase Family 43 protein	GH43
Cphy_3022	Polysaccharide Lyase Family 12 protein	PL12
Cphy_3023	Glycoside Hydrolase Family 29 protein	GH29
Cphy_3028	Glycoside Hydrolase Family 29 protein	GH29
Cphy_3029	Glycoside Hydrolase Family 88 protein	GH88
Cphy_3056	Glycoside Hydrolase Family 36 protein	GH36
Cphy_3069	Carbohydrate Esterase Family 4 protein	CE4
Cphy_3081	Glycoside Hydrolase Family 2 protein	GH2
Cphy_3102	Glycosyltransferase Family 2 protein	GT2
Cphy_3103	Glycosyltransferase Family 2 protein	GT2
Cphy_3109	Glycoside Hydrolase Family 25 protein	GH25
Cphy_3158	Glycoside Hydrolase Family 67 protein	GH67
Cphy_3160	Glycoside Hydrolase Family 2 protein	GH2

Protein ID	Predicted number	CAZy module(s)
Cphy_3202	Glycoside Hydrolase Family 5 / Carbohydrate-Binding Module Family 46 / Carbohydrate-Binding Module Family 2 protein	GH5-CBM46-CBM2
Cphy_3207	Glycoside Hydrolase Family 8 protein	GH8
Cphy_3217	Glycoside Hydrolase Family 28 protein	GH28
Cphy_3239	Glycoside Hydrolase Family 20 protein	GH20
Cphy_3309	Carbohydrate-Binding Module Family 32 protein	CBM32
Cphy_3310	Glycoside Hydrolase Family 28 protein	GH28
Cphy_3313	Glycoside Hydrolase Family 65 protein	GH65
Cphy_3314	Glycoside Hydrolase Family 65 protein	GH65
Cphy_3329	Glycoside Hydrolase Family 3 protein	GH3
Cphy_3367	Glycoside Hydrolase Family 9 / Carbohydrate-Binding Module Family 3 protein	GH9-CBM3-CBM3
Cphy_3368	Glycoside Hydrolase Family 48 / Carbohydrate-Binding Module Family 3 protein	GH48-CBM3
Cphy_3388	Glycoside Hydrolase Family 16 / Carbohydrate-Binding Module Family 4 protein	GH16-CBM4-CBM4-CBM4-CBM4
Cphy_3396	Glycoside Hydrolase Family 4 protein	GH4
Cphy_3398	Glycoside Hydrolase Family 43 protein	GH43
Cphy_3404	Glycoside Hydrolase Family 30 protein	GH30
Cphy_3466	Glycoside Hydrolase Family 73 protein	GH73
Cphy_3501	Glycosyltransferase Family 4 protein	GT4
Cphy_3505	Glycosyltransferase Family 4 protein	GT4
Cphy_3545	Glycosyltransferase Family 4 protein	GT4
Cphy_3571	Glycoside Hydrolase Family 20 protein	GH20
Cphy_3581	Carbohydrate Esterase Family 9 protein	CE9
Cphy_3586	Glycoside Hydrolase Family 53 / Carbohydrate-Binding Module Family 13 protein	GH53-CBM13
Cphy_3618	Glycoside Hydrolase Family 43 protein	GH43
Cphy_3721	Glycosyltransferase Family 2 protein	GT2
Cphy_3727	Glycosyltransferase Family 28 protein	GT28
Cphy_3749	Glycoside Hydrolase Family 18 protein	GH18

Protein ID	Predicted number	CAZy module(s)
Cphy_3785	Glycoside Hydrolase Family 31 protein	GH31
Cphy_3854	Glycoside Hydrolase Family 94 protein	GH94
Cphy_3862	Glycoside Hydrolase Family 10 / Carbohydrate Esterase Family 15 protein	GH10-GH10-CE15
Cphy_3869	Polysaccharide Lyase Family 9 protein	PL9
Cphy_3030	Glycoside Hydrolase Family 112 protein	GH112
Cphy_0577	Glycoside Hydrolase Family 112 protein	GH112
Cphy_1920	Glycoside Hydrolase Family 112 protein	GH112
Cphy_0886	Glycoside Hydrolase Family 113 protein	GH113
Cphy_1719	Glycoside Hydrolase Family 125 protein	GH125
Cphy_0591	Glycoside Hydrolase Family 127 protein	GH127
Cphy_1718	Glycoside Hydrolase Family 130 protein	GH130

[a] Two ORFs yield a complete GT1.

Table B. Genes encoding homologs of carbohydrate-active enzymes (CAZy), whose expression increased during growth on cellulose in comparison to glucose

Protein ID [a]	Predicted function based on CAZy (domain organization) [b]	Fold change in expression during growth on cellulose relative to glucose
**Cphy_1071**	mannanase ([SIGN]-[CBM35]-[GH26]-[LNK]-[CBM3])	10
**Cphy_1510**	xylanase ([GH10])	2
**Cphy_1799**	chitinase ([GH18-CBM12])	77
**Cphy_1800**	chitinase ([CBM12-GH18])	82
**Cphy_2128**	mannanase ([(SIGN]-[CBM35]-[GH26]-[X2]-[X2]-[CBM3])	2
**Cphy_3202**	cellulase ([SIGN]-[GH5]-[X2]-[CBM46]-[LNK]-[CBM2])	3
**Cphy_3367**	cellulase ([SIGN]-[GH9]-[CBM3]-[X2]-[X2]-[CBM3])	33
**Cphy_3368**	processive endo-cellulase ([SIGN]-[GH48]-[X2]-[CBM3])	52

[a] *Genes are organized in ascending order; consecutive numbers indicate close physical proximity.*

[b] *For domain abbreviations see Table A.*

Table C. Genes predicted to be involved in xylan hydrolysis and transport and xylan-dependent gene regulation

Protein ID [a]	Predicted function based on CAZy domain organization or homology to COG database (e-value<0.001) [b]	Fold change in expression during growth on birchwood xylan relative to glucose
**Cphy_1528**	AraC	7
**Cphy_1529**	ABC-type sugar transport system, periplasmic component	113
**Cphy_1530**	ABC-type sugar transport systems, permease component	112
**Cphy_1531**	ABC-type sugar transport system, permease component	105
**Cphy_2105**	Beta-1,4-xylanase ([GH11])	23
**Cphy_2108**	Beta-1,4-xylanase ([SIGN]-[CBM22]-[UNK]-[GH10]-[LNK]-[SORT])	28
**Cphy_2654**	ABC-type sugar transport system, periplasmic component	5
**Cphy_2655**	ABC-type sugar transport system, permease component	17
**Cphy_2656**	ABC-type polysaccharide transport system, permease component	20
**Cphy_3009**	Beta-glucosidase ([GH3])	21
**Cphy_3010**	Beta-1,4-xylanase ([GH10])	18
**Cphy_3206**	Methyl-accepting chemotaxis protein	20
**Cphy_3207**	Xylanase ([GH8])	22
**Cphy_3208**	ABC-type sugar transport system, permease component	22
**Cphy_3209**	ABC-type polysaccharide transport system, permease component	21
**Cphy_3210**	ABC-type sugar transport system, periplasmic component	25
**Cphy_3211**	Response regulator containing CheY-like receiver domain and AraC-type DNA-binding domain	10
**Cphy_3212**	Predicted signal transduction protein with a C-terminal ATPase domain	8

[a] *Genes are organized in ascending order; consecutive numbers indicate close physical proximity.*

[b] *For domain abbreviations see Table A.*

Table D. Genes predicted to be involved in pectin breakdown

Protein ID [a]	Predicted function based on CAZy domain organization or homology to COG database (e-value<0.001)	Fold change in expression during growth on apple pectin relative to glucose
Cphy_0288	glucuronyl hydrolase ([GH88])	3.1
Cphy_0289	ABC-type sugar transport systems, permease component	3.8
Cphy_0290	ABC-type sugar transport systems, permease component	4
Cphy_0291	ABC-type sugar transport system, periplasmic component	2.2
Cphy_0292	putative regulator of cell autolysis	1.8
Cphy_0293	response regulator containing CheY-like receiver domain and AraC-type DNA-binding domain	1.8
Cphy_0339	ABC-type polysaccharide transport system, permease component	3
Cphy_0340	ABC-type sugar transport system, permease component	3
Cphy_0341	ABC-type sugar transport system, permease component	1.4
Cphy_0342	AraC-type DNA-binding domain-containing proteins	2
Cphy_0343	rhamnogalacturonan lyase [(PL11])	1.6
Cphy_1612	pectate lyase ([PL1]-[PL9])	10
Cphy_1714	endo-beta-N-acetylglucosamidase ([GH85]-[CBM32])	3.4
Cphy_1715	binding-protein-dependent transport systems inner membrane component	9.5
Cphy_1716	binding-protein-dependent transport systems inner membrane component	8.6
Cphy_1717	extracellular solute-binding protein family 1	3
Cphy_1718	glycosidase PH1107-related, beta-fructosidase (furanosidase)	4.3
Cphy_1719	uncharacterized conserved protein	4.2
Cphy_1720	alpha-mannosidase ([GH38])	3.9
Cphy_1721	predicted signal transduction protein with a C-terminal ATPase domain	1.4
Cphy_1722	response regulator containing CheY-like receiver domain and AraC-type DNA-binding domain	1.9
Cphy_1723	ABC-type sugar transport system, periplasmic component	1.8

Protein ID [a]	Predicted function based on CAZy domain organization or homology to COG database (e-value<0.001)	Fold change in expression during growth on apple pectin relative to glucose
Cphy_1877	alpha-glucosidase ([GH31])	3
Cphy_1888	pectate lyase ([PL9])	
Cphy_2263	Lysophospholipase L1 and related esterases	8.5
Cphy_2264	glycosidase PH1107-related, beta-fructosidase (furanosidase)	9.5
Cphy_2265	ABC-type sugar transport system, periplasmic component	7.9
Cphy_2266	unknown function	7.8
Cphy_2267	ABC-type sugar transport system, permease component	16
Cphy_2268	ABC-type sugar transport system, permease component	12.7
Cphy_2269	unknown function	16.3
Cphy_2272	ABC-type sugar transport system, permease component	15.7
Cphy_2273	ABC-type sugar transport system, permease component	18.1
Cphy_2274	ABC-type sugar transport system, permease component	9.2
Cphy_2275	predicted integral membrane protein	6.2
Cphy_2276	beta-mannanase ([CBM35]-[GH26])	5.8
Cphy_2919	pectate lyase ([PL9])	6.6
Cphy_3585	transcriptional regulators	18.9
Cphy_3586	arabinogalactan endo-beta-1,4-galactanase ([GH53]-[CBM13])	86
Cphy_3587	unknown function	113.1
Cphy_3588	ABC-type sugar transport system, permease component	78.6
Cphy_3589	ABC-type sugar transport system, permease component	69.2
Cphy_3590	ABC-type sugar transport system, permease component	83.1
Cphy_3869	pectate lyase ([PL9])	2.8

[a] *Genes are organized in ascending order; consecutive numbers indicate close physical proximity.*

[b] *For domain abbreviations see Table A.*

References:

Abbott DW, Boraston AB. 2008. Structural biology of pectin degradation by Enterobacteriaceae. Microbiol. Mol. Biol. Rev. 72:301–316, table of contents. doi: 10.1128/MMBR.00038-07.

Adelsberger H, Hertel C, Glawischnig E, Zverlov VV, Schwarz WH. 2004. Enzyme system of Clostridium stercorarium for hydrolysis of arabinoxylan: reconstitution of the in vivo system from recombinant enzymes. Microbiology (Reading, Engl.). 150:2257–2266. doi: 10.1099/mic.0.27066-0.

Aspinall GO. 1980. Chemistry of cell wall polysaccharides. In: The Biochemistry of Plants.Vol. Preiss, J. p. 473.

Bronnenmeier K, Rucknagel KP, Staudenbauer WL. 1991. Purification and properties of a novel type of exo-1,4-beta-glucanase (avicelase II) from the cellulolytic thermophile *Clostridium stercorarium*. Eur. J. Biochem. 200:379–385.

Charnock SJ et al. 2000. The X6 'Thermostabilizing' domains of xylanases are carbohydrate-binding modules: structure and biochemistry of the *Clostridium thermocellum* X6b domain. Biochemistry. 39:5013–5021. doi: 10.1021/bi992821q.

Davies GJ, Henrissat B. 2002. Structural enzymology of carbohydrate-active enzymes: implications for the post-genomic era. Biochem. Soc. Trans. 30:291–297.

Flint HJ, Bayer EA, Rincon MT, Lamed R, White BA. 2008. Polysaccharide utilization by gut bacteria: potential for new insights from genomic analysis. Nat. Rev. Microbiol. 6:121–131.

Gardy JL et al. 2005. PSORTb v.2.0: expanded prediction of bacterial protein subcellular localization and insights gained from comparative proteome analysis. Bioinformatics. 21:617–623. doi: 10.1093/bioinformatics/bti057.

Garron M-L, Cygler M. 2010. Structural and mechanistic classification of uronic acid-containing polysaccharide lyases. Glycobiology. 20:1547–1573. doi: 10.1093/glycob/cwq122.

Han JS, Rowell JS. 1997. Chemical composition of fibers. In: Paper and composites from agro-based resources. p. 83.

Hehemann J-H et al. 2010. Transfer of carbohydrate-active enzymes from marine bacteria to Japanese gut microbiota. Nature. 464:908–912. doi: 10.1038/nature08937.

Hunter S et al. 2012. InterPro in 2011: new developments in the family and domain prediction database. Nucleic Acids Res. 40:D306–D312. doi: 10.1093/nar/gkr948.

Jauris S et al. 1990. Sequence analysis of the *Clostridium stercorarium celZ* gene encoding a thermoactive cellulase (Avicelase I): identification of catalytic and cellulose-binding domains. Mol. Gen. Genet. 223:258–267.

Kosugi A, Amano Y, Murashima K, Doi RH. 2004. Hydrophilic domains of scaffolding protein CbpA promote glycosyl hydrolase activity and localization of cellulosomes to the cell surface of *Clostridium cellulovorans*. J. Bacteriol. 186:6351–6359. doi: 10.1128/JB.186.19.6351-6359.2004.

Lau J, McNeil M, Darvill A, Albersheim P. 1985. Structure of the backbone of rhamnogalacturonan I, a pectic polysaccharide in the primary cell walls of plants. Carbohydr. Res. 111.

Leschine S. 2005. Degradation of polymers: cellulose, starch, pectin, xylan. In: Handbook on Clostridia. CRC Press: Boca Raton.

Mosbah A et al. 2000. Solution structure of the module X2 1 of unknown function of the cellulosomal scaffolding protein CipC of *Clostridium cellulolyticum*. J. Mol. Biol. 304:201–217. doi: 10.1006/jmbi.2000.4192.

Naumoff DG. 2001. Beta-Fructosidase superfamily: Homology with some alpha-L-arabinases and beta-D-xylosidases. Proteins: Structure, Function, and Genetics. 42:66–76. doi: 10.1002/1097-0134(20010101)42:1<66::AID-PROT70>3.0.CO;2-4.

Pagès S et al. 1999. Sequence analysis of scaffolding protein CipC and ORFXp, a new cohesin-containing protein in *Clostridium cellulolyticum*: comparison of various cohesin domains and subcellular localization of ORFXp. J. Bacteriol. 181:1801–1810.

Reguera G, Leschine S. 2001. Chitin degradation by cellulolytic anaerobes and facultative aerobes from soils and sediments. FEMS Microbiol. Lett. 204:367–374.

Ridley B, O'Neill M, Mohnen D. 2001. Pectins: structure, biosynthesis, and oligogalacturonide-related signaling. Phytochemistry. 57:929–967.

Riedel K, Ritter J, Bronnenmeier K. 1997. Synergistic interaction of the *Clostridium stercorarium* cellulases Avicelase I (CelZ) and Avicelase II (CelY) in the degradation of microcrystalline cellulose. FEMS Microbiol. Lett. 147:239.

Rokas A et al. 2007. What can comparative genomics tell us about species concepts in the genus Aspergillus? Stud. Mycol. 59:11–17. doi: 10.3114/sim.2007.59.02.

Ryttersgaard C et al. 2004. The structure of endo-beta-1,4-galactanase from *Bacillus licheniformis* in complex with two oligosaccharide products. J. Mol. Biol. 341:107–117. doi: 10.1016/j.jmb.2004.05.017.

Schwarz W, Zverlov V, Bahl H. 2004. Extracellular glycosyl hydrolases from clostridia. Adv. Appl. Microbiol. 56:215–261.

Talluri S, Raj SM, Christopher LP. 2013. Consolidated bioprocessing of untreated switchgrass to hydrogen by the extreme thermophile Caldicellulosiruptor saccharolyticus DSM 8903. Bioresource Technology. 139:272–279. doi: 10.1016/j.biortech.2013.04.005.

Tolonen A et al. 2011. Proteome-wide systems analysis of a cellulosic biofuel-producing microbe. Mol. Syst. Biol. 7:461. doi: 10.1038/msb.2010.116.

Tolonen A, Chilaka A, Church G. 2009. Targeted gene inactivation in *Clostridium phytofermentans* shows that cellulose degradation requires the family 9 hydrolase Cphy3367. Mol. Microbiol. 74:1300–1313.

Warnick TA, Methe BA, Leschine S. 2002. Clostridium phytofermentans sp. nov., a cellulolytic mesophile from forest soil. Int J Syst Evol Microbiol. 52:1155–60.

Zhang X et al. 2010. The noncellulosomal family 48 cellobiohydrolase from *Clostridium phytofermentans* ISDg: heterologous expression, characterization, and processivity. Appl. Microbiol. Biotechnol. 86:525–533.
