# Supplementary material for: Genome and Transcriptome of Clostridium phytofermentans, Catalyst for the Direct Conversion of Plant Feedstocks to Fuels
Source: PLoS One. 2015 Jun 2;10(6):e0118285. doi: 10.1371/journal.pone.0118285 (PMC4452783; doi:10.1371/journal.pone.0118285)
Supplement: S6 File — (PDF) [file pone.0118285.s006.pdf]

S6 File. Pentose metabolism.

The pentose sugars, L-arabinose and xylose, are important constituents of hemicellulose and pectin. The metabolism of these sugars by *Clostridium phytofermentans,* was investigated via a combination of comparative genomic and microarray analyses.

Xylose metabolism. The first step in xylose utilization is the uptake of xylose. Due to the large numbers of transporters encoded within the *C. phytofermentans* genome, it was not possible to unambiguously identify a xylose transporter, by comparative genomic analysis. We, therefore, searched the microarray data for transporter genes, which were highly up-regulated in comparison to glucose, only during growth on xylose. We identified an ABC-transporter encoding cluster (Cphy_1585-1587), which was highly up-regulated (8-15 fold relative to glucose) and had a transcript abundance within the 99th percentile during growth on xylose. The solute-binding subunit of this transporter (Cphy_1585) has a high degree of similarity (69%) to the multiple sugar-binding periplasmic receptor of *Agrobacterium tumefaciens*, ChvE, which is thought to bind arabinose, xylose, galactose, glucose, and fucose (Kemner et al. 1997). In fact, Cphy_1585 is highly expressed on a variety of simple sugars with transcript abundances within the 95th percentile. This putative xylose transporter was not upregulated during growth on birchwood xylan, suggesting that *C. phytofermentans* may uptake primarily xylosides during growth on xylan.

Analysis of the genome of *C. phytofermentans* revealed that it is likely to degrade xylose via a commonly used pathway that is found in both xylanolytic and non-xylanolytic bacteria, conversion of xylose to D-xylulose 5-phosphate followed by entry into the reductive branch of the pentose phosphate pathway (Figure S2). All of the enzymes within the putative xylose pathway of *C. phytofermentans* share at least 60% similarity with characterized enzymes (Table A). Comparison of global gene expression during growth on xylose and xylan to growth on glucose further supported the presence of this pathway. The enzymes involved in the conversion of xylose to xylulose-5-phosphate, two xylose isomerases (Cphy_0200 and Cphy_1219) and a xylulokinase (Cphy_3419), were up-regulated 6-15 fold during growth on xylose and xylan, reaching transcript abundances within the 90th percentile (Table A). In contrast, expression of the genes within the pentose phosphate pathway was relatively constant, consistent with their central metabolic role.

A recent comparative genomic study of xylose utilization in the firmicutes (Gu et al. 2010) suggested that, in *C. phytofermentans,* the xylose isomerase and xylose kinase in conjunction with a GH43 xylanase (XynB, Cphy_2632) and a transcriptional repressor with a sugar kinase-like domain, XylR (Cphy_3420) constitute part of a regulon controlled by XylR. Both the GH43 xylanase and XylR were up-regulated during growth on xylose and xylan relative to glucose, but not as dramatically as the xylose-degrading genes described above. Expression of the xylanase increased 1.6 and 5 fold on xylose and xylan, respectively, and expression of XylR, increased 3 and 6 fold on xylose and xylan, respectively. With the exception of growth on xylose, xylan and arabinose, XylR

transcript abundance was consistently very low, within the 20th percentile. These results are consistent with XylR playing a role in regulating pentose metabolism and suggest that further investigation of the role of XylR in *C. phytofermentans* is warranted.

L-arabinose metabolism. Although the combination of genomic and microarray analysis yielded useful insights into the utilization of xylose by *C. phytofermentans*, this was not the case for the metabolism of L-arabinose. Culturing of *C. phytofermentans* in arabinose induced the expression of multiple groups of genes that were also differentially expressed during growth on other substrates, including a group of cellulases which have no clear direct role in arabinose metabolism and various genes implicated in the uptake and metabolism of xylose (Table A).

To date four distinct pathways for L-arabinose metabolism have been identified and genes encoding the relevant enzymes have been identified for two of them (reviewed in Watanabe 2005). Using comparative genomic analysis, we were only able to identify one gene, Cphy_0584, which had limited similarity (33%) to the arabinose isomerase (AraA) of *Bacillus halodurans* (Dong-Woo Lee et al. 2005). AraA catalyzes the first step in the most common bacterial L-arabinose degradation pathway, which consists of an isomerase (AraA), a kinase (AraB), and an epimerase (AraD) that sequentially convert L-arabinose to L-ribulose, L-ribulose 5-phosphate, and D-xylulose 5-phosphate. Cphy_0584 is found within a cluster that also encodes an ABC-transporter (Cphy_0580-0584), which is upregulated 3-6.5 fold during growth on arabinose (Table A). However, no AraB or AraD homologs are present in the genome. Thus, the mechanism of arabinose degradation by *C. phytofermentans* remains unknown. It may be unusual and

its identification may be hindered by the induction of multiple genes with functions unrelated to arabinose metabolism during growth in the presence of L-arabinose. The complexity of the transcriptional response to arabinose may reflect the fact that in nature, L-arabinose is rarely found in its free form, and is instead a constituent of complex polymers.

Table A. Genes involved in pentose metabolism

Protein ID	Predicted protein function	Transcript abundance percentile (Fold change in expression relative to glucose)					Closest characterized homolog		
		glucose	xylose	arabinose	xylan	cellulose	Organism	Protein (% similarity [a])	Reference
Cphy_1585	solute-binding component of ABC transporter	95	99 (8)	98 (3)	64 (-2)	94 (1)	*Agrobacterium tumefaciens*	ChvE (68.6)	(Kemner et al. 1997)
Cphy_1586	ABC transporter related	58	99 (11)	92 (3)	60 (-2)	80 (1)	*A. tumefaciens*	XylG (79.5)	(Kemner et al. 1997)
Cphy_1587	monosaccharide-transporting ATPase	73	99 (15)	92 (4)	64 (1)	83 (2)	*A. tumefaciens*	XylH (63.5)	(Kemner et al. 1997)
Cphy_0200	xylose isomerase	49	96 (10)	98 (19)	97 (15)	64 (1)	*Clostridium acetobutylicum*	XylA-II (85.2)	(Gu et al. 2010)
Cphy_1219	xylose isomerase	76	98 (9)	99 (18)	99 (13)	76 (1)	*Bacillus subtilis*	XylA (71.3)	(Wilhelm & Hollenberg 1985)
Cphy_3419	xylulokinase	28	85 (5)	89 (6)	94 (11)	48 (1)	*C. acetobutylicum*	XylB (60.9)	(Gu et al. 2010)
Cphy_3420	xylR (ROK family protein)	14	35 (2)	49 (3)	61 (6)	19 (1)	*C. acetobutylicum*	XylR (47.3)	(Gu et al. 2010)
Cphy_2484	ribulose-phosphate 3-epimerase	76	74 (-2)	65 (-3)	84 (1)	71 (-2)	*Escherichia coli*	Rpe (68.9)	(Lyngstadaas et al. 1998)
Cphy_3745	ribose-phoshate isomerase	90	83 (-2)	81 (-2)	89 (1)	86 (-2)	*E. coli*	RpiB (61.3)	(Sorensen & Hove-Jensen 1996)
Cphy_0013	transaldolase	82	82 (-2)	83 (-2)	96 (2)	77 (-2)	*Methanococcus jannaschii*	Tal (75.8)	(Soderberg & Alver 2004)
Cphy_0014	transketolase	87	88 (-2)	87 (-2)	97 (2)	81 (-2)	*B. subtilis*	Tkt (69.2)	(De Wulf et al. 1997)
Cphy_2632	glycoside hydrolase family 43	35	50 (1)	63 (2)	82 (4)	31 (-2)	*Geobacillus stearothermophilus*	XynB (40.5)	(Brüx et al. 2006)
Cphy_0580	ABC transporter related	8	11 (1)	43 (3)	14 (-2)	10 (1)	*Rhizobium leguminosarum*	RhaT (62.2)	(Richardson et al. 2004)
Cphy_0581	monosaccharide-transporting ATPase	8	7 (-2)	43 (4)	15 (1)	14 (1)	*R. leguminosarum*	RhaP (57.1)	(Richardson et al. 2004)
Cphy_0582	monosaccharide-transporting ATPase	14	14 (1)	63 (6)	18 (1)	19 (1)	*R. leguminosarum*	RhaQ (56.6)	(Richardson et al. 2004)

Protein ID	Predicted protein function	Transcript abundance percentile (Fold change in expression relative to glucose)					Closest characterized homolog		
Cphy_0583	putative sugar ABC transporter, substrate-binding protein	7	11 (1)	47 (4)	18 (1)	6 (1)	*R. leguminosarum*	RhaS (53.0)	(Richardson et al. 2004)
Cphy_0584	L-arabinose isomerase	8	10 (1)	35 (3)	11 (-2)	6 (1)	*Bacillus halodurans*	AraA (32.6)	(Dong-Woo Lee et al. 2005)

[a] *All percent similarity values were derived from pairwise global alignments of protein sequences generated using the algorithm of Needleman and Wunsch* (1970).

References:

Brüx C et al. 2006. The structure of an inverting GH43 beta-xylosidase from *Geobacillus stearothermophilus* wth its substrate reveals the role of the three catalytic residues. J. Mol. Biol. 359:97-109.

Gu Y et al. 2010. Reconstruction of xylose utilization pathway and regulons in Firmicutes. BMC Genomics. 11:255.

Kemner JM, Liang X, Nester EW. 1997. The *Agrobacterium tumefaciens* virulence gene chvE is part of a putative ABC-type sugar transport operon. J. Bacteriol. 179:2452-2458.

Lee D-W et al. 2005. Distinct metal dependence for catalytic and structural functions in the L-arabinose isomerases from the mesophilic *Bacillus halodurans* and the thermophilic *Geobacillus stearothermophilus*. Arch. Biochem. Biophys. 434:333-343.

Lyngstadaas A, Sprenger GA, Boye E. 1998. Impaired growth of an *Escherichia coli* rpe mutant lacking ribulose-5-phosphate epimerase activity. Biochim. Biophys. Acta. 1381:319–30.

Needleman S, Wunsch C. 1970. A general method applicable to the search for similarities in the amino acid sequence of two proteins. J. Mol. Biol. 48:443-453.

Richardson J, Hynes M, Oresnik I. 2004. A genetic locus necessary for rhamnose uptake and catabolism in *Rhizobium leguminosarum* bv. *trifolii*. J. Bacteriol. 186:8433-8442.

Soderberg T, Alver RC. 2004. Transaldolase of *Methanocaldococcus jannaschii*. Archaea. 1:255-62.

Sorensen KI, Hove-Jensen B. 1996. Ribose catabolism of *Escherichia coli*: characterization of the rpiB gene encoding ribose phosphate isomerase B and of the rpiR gene, which is involved in regulation of rpiB expression. J. Bacteriol. 178:1003-11.

Watanabe S. 2005. Cloning, expression, and characterization of bacterial L-arabinose 1-dehydrogenase involved in an alternative pathway of L-arabinose metabolism. J. Biol. Chem. 281:2612-2623.

Wilhelm M, Hollenberg CP. 1985. Nucleotide sequence of the *Bacillus subtilis* xylose isomerase gene: extensive homology between the *Bacillus* and *Escherichia coli* enzyme. Nucleic Acids Res. 13:5717-22.

De Wulf P, Soetaert W, Schwengers D, Vandamme EJ. 1997. Optimization of D-ribose production with a transketolase-affected *Bacillus subtilis* mutant strain in glucose and gluconic acid-based media. J. Appl. Microbiol. 83:25-30.
