# Supplementary material for: Genome and Transcriptome of Clostridium phytofermentans, Catalyst for the Direct Conversion of Plant Feedstocks to Fuels
Source: PLoS One. 2015 Jun 2;10(6):e0118285. doi: 10.1371/journal.pone.0118285 (PMC4452783; doi:10.1371/journal.pone.0118285)
Supplement: S7 File — (PDF) [file pone.0118285.s007.pdf]

S7 File. Investigation of *C. phytofermentans* central metabolism: Insights into high levels of ethanol production.

Introduction

Perhaps the most industrially desirable property of *C. phytofermentans* is that it produces ethanol as the major fermentation product during growth on a wide variety of substrates including simple sugars, cellulose and minimally processed plant feedstocks (This study and Warnick et al., 2002). As the conversion of acetyl-CoA to ethanol results in the oxidation of two molecules of NADH to NAD+, ethanol production is generally thought to play a key role in the maintenance of intracellular redox balance and in the regeneration of NAD+ for the glycolytic pathway. Conversion of acetyl-CoA to acetate, a competing fermentation product, generates ATP by substrate-level phosphorylation. Thus, production of high levels of ethanol by *C. phytofermentans*, suggests that it can meet its energy (ATP) requirements while still producing high levels of ethanol. In this section, we try to gain insight into the basis of the high levels of ethanol synthesis by *C. phytofermentans* by identifying central metabolic genes that are highly expressed on all growth substrates. The microarray data were therefore searched for highly expressed genes (average transcript abundance within the 90th percentile) that could be assigned functions related to ethanol production, energy generation, or the maintenance of redox balance. The results of this analysis were used to construct a preliminary model of *C. phytofermentans* central metabolism (Figure 6, Table A). The expression levels of many of the genes identified in the present analysis are in agreement with proteomic analysis of *C. phytofermentans* growing on glucose, hemicellulose, and cellulose (Tolonen et al., 2011).

Glycolytic and ethanol biosynthesis pathways of C. *phytofermentans*: Transcriptional evidence for funneling of pyruvate to ethanol.

Analysis of the genome indicates that *C. phytofermentans* most likely metabolizes hexose sugars and derivatives of pentose sugars to pyruvate via the Embden-Meyerhof-Parnas glycolytic pathway (Figure 6, Table A). One noticeable feature of the glycolytic pathway of *C. phytofermentans* that may reduce the need for acetate-dependent ATP synthesis, is that there are two steps that can be catalyzed by either ATP- or pyrophosphate-dependent enzymes: the phosphorylation of fructose-6-phosphate and the conversion of phosphoenolpyruvate (PEP) to pyruvate. Exclusive use of pyrophosphate-dependent glycolytic enzymes in conjunction with adenylate kinase can increase the ATP yield of glycolysis 2.5 fold (Mertens, 1993). Analysis of the microarray data indicates that the adenylate kinase homolog (Cphy_3646, Table A) was constitutively highly expressed (98th percentile). The homologs of ATP-(Cphy_0336, Table A) and pyrophosphate-(Cphy_3345, Table A) dependent phosphofructokinases have similar transcript levels suggesting that both are utilized for the synthesis of fructose-bisphosphate. In contrast, pyruvate synthesis from phosphoenol pyruvate, an ATP-producing reaction, appears to be primarily pyrophosphate-dependent. The pyruvate phosphate dikinase homolog (Cphy_0651) is one of the most highly expressed genes in the genome (100th percentile) and its transcripts are far more abundant than those of its ATP-dependent equivalents, homologs of pyruvate kinases (Cphy_0741 and Cphy_2900) (Table A). Thus, the utilization of the most energetically efficient glycolytic enzymes (pyrophosphate- rather than ATP- dependent) by *C. phytofermentans* may contribute to ethanol production, indirectly, by increasing the ATP yield of glycolysis and

consequently, decreasing the demand for ATP generated from the production of the competing fermentation product, acetate.

Microarray analysis revealed a potentially surprising feature of the glycolytic pathway of *C. phytofermentans* – there appears to a high level of flux into or out of the pathway at phosphoenol pyruvate (PEP). One of the most highly expressed genes in the genome (transcript abundance within the 100th percentile) is an unusual PEP carboxykinase (Pepck, Cphy_3853, Table A). Only one homolog of Cphy_3853 (68% similar) has been characterized, the Pepck of *Streptococcus equinus* (Narito Asanuma et al., 2010). The *S. equinus* enzyme is monodirectional, and catalyzes the decarboxylation of oxaloacetate to yield PEP utilizing either ATP or GTP. If the *C. phytofermentans* Pepck is also monodirectional, any excess oxaloacetate derived from the catabolism of amino acids (see below) may be funneled into the ethanol biosynthetic pathway via its conversion to PEP (Figure 6). However, this is an ATP-consuming reaction. Another possibility is that Pepck of *C. phytofermentans* may operate in the opposite direction and synthesize both ATP and oxaloacetate from PEP and CO_2, which could serve both as a precursor for amino acid biosynthesis and as a substrate for the sodium-translocating decarboxylase Cphy_2433-2437 (see below).

The first step in the production of ethanol from the end product of glycolysis, pyruvate, is the conversion of pyruvate to acetyl-CoA (Fig. S2). *C. phytofermentans* appears to convert pyruvate to acetyl-CoA primarily using a highly expressed pyruvate ferredoxin oxidoreductase (PFOR, Cphy_3558, Table A), which catalyzes the decarboxylation of pyruvate to acetyl-CoA along with reduction of ferredoxin. Cphy_3558 is highly similar (>70%) to several biochemically characterized, monomeric

PFOR including that of *Clostridium acetobutylicum* (Meinecke et al., 1989). Genes encoding other enzymes involved in pyruvate consumption: lactate dehydrogenase (LDH) (Cphy_1232 and Cphy_1117) and an unusual multi-subunit pyruvate:formate lyase (PFL) (Cphy_2820-2823) were, on average, expressed at moderate or low levels (Table A). This suggests that in *C. phytofermentans*, the majority of pyruvate, that is not diverted to biosynthetic processes, is converted to acetyl-CoA with concomitant reduction of ferredoxin. This prediction is consistent with fermentation product profiles –derivatives of acetyl-CoA, ethanol and acetate, dominate, whereas formate and lactate are generally minor products.

Conversion of acetyl-CoA to ethanol involves two reduction reactions, carried out sequentially by acetaldehyde (ALD) and alcohol (ADH) dehydrogenases, which simultaneously oxidize either NADH or NADPH. Two ADH, Cphy_3925 and Cphy_1029, were constitutively expressed at extremely high levels that rivaled or exceeded those of many ribosomal proteins (average transcript abundances within the 100th percentile, Table A). Based on its high degree of similarity (74%) to AdhE of *Escherichia coli* (Kessler et al., 1991), Cphy_3925 is likely to be a bifunctional ALD/ADH, which catalyzes the sequential, NADH-dependent, conversion of acetyl-CoA to acetaldehyde to ethanol. In contrast, Cphy_1029 is predicted to be a monofunctional NADPH-dependent ADH, based on its similarity (88%) to the NADPH-dependent butanol and ethanol dehydrogenase of *Clostridium saccharobutylicum* (Youngleson et al., 1989). A third monofunctional NADH-dependent ADH, Cphy_2463, (75% similar to the NADH-dependent ADH of *C. acetobutylicum* (Walter et al., 1992)), was also constitutively highly expressed, although at lower levels than the above-mentioned

ALD/ADH and NADPH-dependent ADH (Table A). We were unable to identify any monofunctional ALDs that were expressed at levels approaching those of the monofunctional NADPH-dependent ADH or the ALD/ADH. The most highly expressed monofunctional ALD, Cphy_3041, was only moderately expressed (average transcript abundance within the 70th percentile). This suggests that *C. phytofermentans* may express an unusual ALD that is difficult to identify by comparative genomic analysis or the bifunctional acetaldehyde/ethanol dehydrogenase (Cphy_3925) may also provide acetaldehyde for Cphy_1029.

In addition to serving as a precursor for ethanol biosynthesis, acetyl-CoA can also be converted to the competing fermentation product, acetate. Conversion of acetyl-CoA to acetate results in the synthesis of ATP via substrate level phosphorylation and is catalyzed by phosphate acetyltransferase (Cphy_1326) and acetate kinase (Cphy_1327). Although these genes were highly and moderately expressed, respectively, both had transcript levels (94th and 77th percentile, respectively) that were significantly lower than either of the two most highly expressed ADHs (~100th percentile).

Acetyl-CoA can also be consumed during the production of amino acid precursors or the breakdown of amino acid catabolites via the TCA cycle. The TCA cycle of *C. phytofermentans* is not complete. Although, enzymes for the initial TCA cycle reactions, from condensation of acetyl-CoA with oxaloacetate to the formation of succinyl-CoA are present, *C. phytofermentans* appears to be missing succinyl-CoA synthetase and malate dehydrogenase. Two of the enzymes of the TCA cycle of *C. phytofermentans* differ from those present in *E. coli* and *B. subtilis. C. phytofermentans* may produce 2-oxoglutarate with an NADH-generating, rather than an NADPH-generating, isocitrate dehydrogenase

(Cphy_3096) and 2-oxoglutarate is likely to be converted to succinyl-CoA by a highly expressed 2-oxoglutarate ferredoxin oxidoreductase (OOR) (Cphy_3122-3), instead of an NADH-generating 2-oxoglutarate dehydrogenase. Oxoglutarate oxidoreductase activity has been detected in *C. acetobutylicum* by ^{13}C flux analysis (Amador-Noguez et al., 2010), indicating that clostridia may use OOR instead of 2-oxoglutarate dehydrogenase. Cphy_3122 and Cphy_3123 are >73% similar to the two subunits of the putative OOR of *Clostridium acetobutylicum.*

Transcriptional evidence for the contribution of pathways for the interconversion and dissipation of ferredoxin-reducing equivalents to ethanol biosynthesis and energy production.

Because conversion of pyruvate to acetyl-CoA is coupled to ferredoxin reduction, it is likely that ferredoxin metabolism plays an important role in ethanol biosynthesis. The microarray data were, therefore, searched for highly expressed genes that could be translated into proteins, which might form complexes that dissipate excess ferredoxin-reducing equivalents (critical for sustaining acetyl-CoA production), or in transferring electrons from ferredoxin to NAD or NADP, such that they could contribute to ethanol production.

1. Hydrogenases. Hydrogenases are thought to play a major role in dissipating ferredoxin-reducing equivalents and maintaining intracellular redox balance in clostridia (R. K. Thauer et al., 1977; M. W. Adams et al., 1980). *C. phytofermentans* generates free hydrogen as a product of the fermentation of cellulose and cellobiose (Warnick et al., 2002; Ren et al., 2007) and the genome of *C. phytofermentans* encodes as many as six distinct hydrogenases, two potential hydrogenase-related signaling complexes, and a

variety of putative hydrogenase maturation proteins (Table A). Three putative cytoplasmic [FeFe]-hydrogenase-encoding clusters were constitutively expressed at high levels (Cphy_0086-0089, Cphy_0090-0093, and Cphy_3801-3805) (Table A). Although further investigation is clearly necessary, we propose that these three hydrogenases may enable *C. phytofermentans* to utilize hydrogen both for eliminating excess reducing equivalents and as an intermediate in the transfer of electrons from ferredoxin to NADH, which can subsequently contribute to ethanol production (Figure 6).

Analysis of expression of the intergenic regions of Cphy_0090-0093 and Cphy_0086-0089 indicates that they constitute two independent operons. The cluster comprising Cphy_0090-0093 may play an important role in ferredoxin-dependent hydrogen production. This cluster is homologous to the *hfs* operon of *Thermoanaerobacterium saccharolyticum* (60-68% similarity in amino acid sequence for the four gene products, Table A), which, when deleted, decreased hydrogen production by *T. saccharolyticum* by 95% (Shaw et al., 2009). The *hfs* cluster has been proposed to both regulate and catalyze ferredoxin-dependent hydrogen production and encodes a protein of unknown function (Cphy_0093 with 62% similarity to *hfsA*), an RbsU regulatory phosphatase (Cphy_0091 with 65% similarity to *hfsC*) and two proteins with [FeFe]-hydrogenase catalytic (H-cluster) domains (Cphy_0090 and Cphy_0092 with 60% similarity to *hfsD* and *hfsB*, respectively), one of which (*hfsB*) also contains a redox-sensing PAS-domain.

Cphy_0087-0089 may be involved in hydrogen-dependent NAD reduction. Its three subunits are 75-83% similar to those of *HupCBA* of *Clostridium saccharoperbutylacetonicum* (Table A), which has been proposed to function as an NAD-

reducing uptake hydrogenase, based on its sequence, expression pattern, and the finding that inhibiting its expression increases hydrogen accumulation (Nakayama et al., 2008). The cluster comprising Cphy_3801-3805, may also contribute to hydrogen-dependent NADH production. Its products are homologous (61-82% similar, Table A) to those of the *hyd* operon of *Thermoanaerobacter tengcongensis*, which encodes a heterotetramic NADH-dependent [FeFe]-hydrogenase involved in hydrogen production (Soboh et al., 2004). The finding that addition of hydrogen to the headspace of cultures of *C. phytofermentans* increased the ethanol to acetate ratio (Shaw et al., 2009) is consistent with the presence of NADH-producing uptake hydrogenases in *C. phytofermentans*. In previous studies (G. Schut & M. Adams, 2009; Calusinska et al., 2010; Tolonen et al., 2011), both Cphy_0087-0089 and Cphy_3801-3805 were proposed to function as bifurcating hydrogenases that produce hydrogen via the oxidation of both ferredoxin and NADH based on their homology to that of *Thermotoga maritima* (G. J. Schut & M. W. W. Adams, 2009). However they are significantly less similar to this enzyme than to the homologs described above.

Although further investigation is clearly necessary, the combination of a ferredoxin-oxidizing, hydrogen-producing hydrogenase encoded by the *hfs* cluster with an NAD-reducing uptake hydrogenase may potentially serve as a pathway for the conversion of ferredoxin to NADH in *C. phytofermentans* (Figure 6). Such a pathway would enable ferredoxin reducing equivalents to contribute to ethanol production and could also potentially help *C. phytofermentans* to maintain a low hydrogen partial pressure and thus a high rate of ferredoxin turnover.

2. NADH and ferredoxin-dependent generation of NADPH. *C. phytofermentans* was found to constitutively express a homolog of the NADH-dependent reduced ferredoxin:NADP oxidoreductase of *Clostridium* at high levels (Cphy_2934-2935, 82 to 84% similar to NfnB and NfnA of *Clostridium kluyveri*) (Wang et al., 2010) at high levels. NfnAB may be a major source of NADPH for *C. phytofermentans*. The only other highly expressed pathway for the production of NADPH that could be identified in the genome was a non-phosphorylating NADP-dependent glyceraldehyde-3-phosphate dehydrogenase (Cphy_2418, 73% similar to GAPDH, Table A). This enzyme serves as an important source of NADPH in *Streptococcus equinus* (Narito Asanuma et al., 2010). The genome of *C. phytofermentans* does not encode two other common NADPH-generating pathways, transhydrogenase and the oxidative branch of the pentose phosphate pathway.

3. Ferredoxin-dependent generation of NADH and ATP. Further examination of the genome sequence and microarray data revealed a pathway whereby *C. phytofermentans* can utilize reduced ferredoxin to generate both NADH, an electron donor for ethanol production, and ATP, which could contribute indirectly to ethanol production by decreasing the demand for ATP derived from acetate production (Figure 6). An Rnf-type NADH:ferredoxin oxidoreductase (Cphy_0211-0216, 44-61% similar to the Rnf of *Acetobacterium woodii*, Table A) and a sodium-translocating F_1F_o-ATPase (Cphy_3735-3742, 43-86% similar to the ATPase of *Clostridium paradoxum*, Table A) were both constitutively expressed at high levels (Table A). Rnf is a membrane-bound complex that can exploit the energy difference between reduced-ferredoxin and NADH to translocate sodium, thereby creating an electrochemical gradient that can drive ATP production by

the F_1F_o-ATPase (reviewed in Biegel et al., 2011). The sodium specificity of the F_1F_o-ATPase of *C. phytofermentans* was deduced based on the amino acid sequence of its C subunit, which contains conserved residues critical for sodium translocation and is highly similar to the C-subunits of the characterized sodium-translocating, energy-producing F_1F_o-ATPase of *Clostridium paradoxum* (Ferguson et al., 2006).

Additional investigation of the role of sodium in *C. phytofermentans* bioenergetics.

The abundance of the transcripts for the sodium-translocating F_1F_o-ATPase, suggested that the sodium electrochemical gradient might play a central role in *C. phytofermentans* physiology. We, therefore, searched for additional constitutively expressed clusters that could be involved either in the generation or maintenance of the sodium electrochemical gradient. A putative sodium-translocating decarboxylase (Cphy_2433-2437, 37.8-67.2 % similar to the decarboxylase of *Veillonella parvula* and *Klebsiella pneumoniae*) was constitutively expressed at high levels (Table A). The cluster encoding the decarboxylase includes a putative sodium-translocating beta subunit (Cphy_2434,), a putative biotin-binding gamma subunit (Cphy_2435), a putative hydrophobic delta subunit (Cphy_2436) and two alpha subunits (Cphy_2433 and Cphy_2437), which are thought to confer substrate specificity (Buckel, 2001; Huder & Dimroth, 1993). One of the putative alpha subunits (Cphy_2433) is most similar to that of the oxaloacetate decarboxylases of *K. pneumoniae*, whereas the other (Cphy_2437) is most similar to those of methyl-malonyl-CoA decarboxylases of *V. parvula* (Table A). Although further investigation will be required to elucidate the substrate and physiological role of this complex, the observation that it is highly expressed suggests that it may be contributing to sodium translocation.

We also identified a moderately expressed cluster encoding a homolog of the ATP-consuming, sodium-extruding V-type-ATPases of *Caloramator fervidus* (Cphy_3070-3077, 39-88% similar, Table A) (Ubbink-Kok et al., 2006), which has been implicated in maintenance of the sodium electrochemical gradient during growth in alkaline conditions. Simultaneous expression of this V-type ATPase and the F_1F_o-ATPase may enable *C. phytofermentans* to finely tune its electrochemical sodium gradient and grow at alkaline pH. It is possible that the sodium-pumping V-type ATPase may also participate in energy production. Interestingly, a second V-type ATPase cluster (Cphy_3440-3447, Table A), which is not highly similar to any characterized complexes and is found in multiple clostridia belonging to Cluster XIVa, is either moderately or highly expressed on most of the growth substrates tested.

Conclusions

Examination of the genomes of several well-studied cellulolytic and solventogenic clostridia indicated that with the exception of the unusual Pepck, the sodium-translocating F_1F_o-ATPase and the sodium-translocating decarboxylase, none of the enzymes and complexes discussed above are unique to *C. phytofermentans*. It may therefore be the specific combination of enzymes and their transcriptional regulation that makes *C. phytofermentans* metabolism unique.

In summary, genomic analysis and transcriptional profiling suggest that high levels of ethanol production by *C. phytofermentans* may be due to a combination of factors. These include: 1) increasing the energetic yield of glycolysis by utilizing pyrophosphate-dependent enzymes; 2) high levels of expression of the enzymes involved in ethanol production coupled with the ability to utilize both NADH and NADPH for

ethanol biosynthesis; 3) the presence of multiple pathways for the dissipation of excess reducing equivalents; and 4) the presence of sodium-dependent energy generating pathways. Experimental studies will be required to determine if these hypotheses are indeed valid.

Table A. Genes involved in central metabolism and high levels of ethanol production.

Protein ID	Predicted protein function	Average transcript abundance percentile (over all substrates)	Closest characterized functional homolog		
			Organism	Protein (% similarity [a])	Reference [b]
			**Glycolysis**		
**Cphy_0329**	glucokinase	89	*Bacillus subtilis* strain 168	GlcK (61)	(Skarlatos & M. Dahl, 1998)
**Cphy_0336**	atp-dependent 6-phosphofructokinase	76	*Clostridium acetobutylicum* ATCC 824	PfkA (73)	(Belouski et al., 1998)
**Cphy_0419**	glucose-6-phosphate isomerase	76	*B. subtilis* strain 168	Pgi (80)	(Prasad et al., 1972)
**Cphy_0651**	pyruvate phosphate dikinase	100	*Clostridium symbiosum* WAL-14163	Ppdk (90)	(Pocalyko et al., 1990)
**Cphy_0741**	pyruvate kinase	70	*B. subtilis* strain 168	PykA (68)	(Muñoz et al., 1997)
**Cphy_2418**	non-phosphorylating NADP-dependent glyceraldehyde-3-phosphate dehydrogenase	92	*Streptococcus bovis*	Gapn (72)	(N. Asanuma & T. Hino, 2006)
**Cphy_2868**	2,3-bisphosphoglycerate-independent phosphoglycerate mutase	92	*B. subtilis* strain 168	GpmI (72)	(Leyva-Vazquez & Setlow, 1994)
**Cphy_2874**	triose phosphate isomerase	89	*Escherichia coli* O157:H7 str. EDL933	TpiA (56)	(Pichersky et al., 1984)
**Cphy_2875**	phosphoglycerate kinase	97	*E. coli* O157:H7 strain EDL933	Pgk (64)	(Alefounder & Perham, 1989)

Protein ID	Predicted protein function	Average transcript abundance percentile (over all substrates)	Closest characterized functional homolog		
**Cphy_2876**	glyceraldehyde-3-phosphate dehydrogenase	99	*C. acetobutylicum* ATCC 824	Gapdh (81)	(Schreiber & Durre, 1999)
**Cphy_2900**	pyruvate kinase	84	*E. coli* O157:H7 str. EDL933	PykF(65)	(Ohara et al., 1989; Ponce et al., 1995)
**Cphy_3001**	enolase (2-phosphoglycerate dehydratase)	89	*B. subtilis* strain 168	Eno (76)	(Leyva-Vazquez & Setlow, 1994)
**Cphy_3345**	pyrophosphate dependent 6-phosphofructokinase	67	*Methylococcus capsulatus* str. Bath	Pfp (53)	(Reshetnikov et al., 2008)
**Cphy_3345**	pyrophosphate-dependent 6-phosphofructokinase	67	*M. capsulatus* strain Bath	Pfp (53)	(Reshetnikov et al., 2008)
**Cphy_3646**	adenylate kinase	98	*B. subtilis* strain 168	Adk (72)	(Bae & Phillips, 2004)
**Cphy_3683**	fructose-1,6-bisphosphate aldolase	97	*B. subtilis* strain 168	FbaA (68)	(Mitchell et al., 1992)
**Mixed acid fermentation**					
**Cphy_1029**	NADPH-dependent alchohol dehydrogenase	100	*Clostridium saccharobutylicum*	Adh1 (88)	(Youngleson et al., 1989)
**Cphy_1117**	lactate dehydrogenase	23	*Lactococcus lactis* subsp. *lactis*	LdhA (59)	(Llanos et al., 1992)
**Cphy_1232**	lactate dehydrogenase	49	*Lactococcus lactis* subsp. *lactis*	LdhA (55)	(Llanos et al., 1992)
**Cphy_1326**	phosphate acetyltransferase	94	*Methanosarcina thermophila*	Pta (75)	(Latimer & Ferry, 1993)

**Protein ID**	**Predicted protein function**	**Average transcript abundance percentile (over all substrates)**	**Closest characterized functional homolog**		
**Cphy_1327**	acetate kinase	77	*C. acetobutylicum*	AckA (75)	(Winzer et al., 1997)
**Cphy_2463**	NADH-dependent alcohol dehydrogenase	91	*C. acetobutylicum*	BdhA (74)	(Walter et al., 1992)
**Cphy_2820**	formate acetyltransferase activating enzyme	84	*E. coli* O157:H7 strain EDL933	PflA (65)	(Rödel et al., 1988)
**Cphy_2821**	formate acetyltransferase	26	*Staphylococcus aureus* subsp. *aureus* MSHR113	PflB (89)	(Leibig et al., 2011)
**Cphy_2822**	rnr-pfl superfamily	66	na		na
**Cphy_2823**	formate acetyltransferase	80	*S. aureus* subsp. *aureus* MSHR1132	PflB (75)	(Leibig et al., 2011)
**Cphy_3558**	pyruvate ferredoxin oxidoreductase	97	*C. acetobutylicum* ATCC 824	Por (72)	(Meinecke et al., 1989)
**Cphy_3925**	bifunctional NADH-dependent acetaldehyde-CoA/alcohol dehydrogenase	100	*E. coli*	AdhE (73)	(Kessler et al., 1991)
**Unknown: mixed acid fermentation or TCA cycle/amino acid biosynthesis**					
**Cphy_3096**	NADH-generating isocitrate dehydrogenase	73	*E. coli*	Icd (44)	(Kabir & Shimizu, 2004)
**Cphy_3122**	pyruvate ferredoxin oxidoreductase alpha subunit	97	*Halobacterium sp.* NRC-1	PorB (50)	(Plaga et al., 1992)
	2-oxoglutarate ferredoxin oxidoreductase alpha subunit		*Halobacterium sp.* NRC-1	KorB (55)	(Ng et al., 2000)

Protein ID	Predicted protein function	Average transcript abundance percentile (over all substrates)	Closest characterized functional homolog		
	2-oxoglutarate ferredoxin oxidoreductase subunit beta		*C. acetobutylicum* ATCC 824	KorB (78)	na
**Cphy_3123**	pyruvate ferredoxin oxidoreductase beta subunit	95	*Halobacterium sp.* NRC-1	PorA (50)	(Plaga et al., 1992)
	2-oxoglutarate ferredoxin oxidoreductase beta subunit		*Halobacterium sp.* NRC-1	KorA (52)	(Plaga et al., 1992)
	2-oxoacid:ferredoxin oxidoreductase		*C. acetobutylicum* ATCC 824	KorA (73)	na
**Decarboxylation**					
**Cphy_3853**	phosphoenolpyruvate carboxykinase	100	*Streptococcus equinus*	Pepck (68)	(Narito Asanuma et al., 2010)
**Hydrogen metabolism/interconversion of reducing equivalents**					
**Cphy_0086**	hypothetical protein	96	*T. saccharolyticum*	Hfsa (68)	(Shaw et al., 2009)
**Cphy_0087**	NADH-dependent [FeFe] uptake hydrogenase, catalytic subunit	97	*Clostridium saccharoperbutylacetonicum* ATCC 27021	HupA (76)	(Nakayama et al., 2008)
**Cphy_0088**	NADH-dependent [FeFe] uptakehydrogenase, FMN- and NAD-binding diaphorase subunit	100	*C. saccharoperbutylacetonicum* ATCC 27021	HupA (83)	(Nakayama et al., 2008)
**Cphy_0089**	NADH-dependent [FeFe] uptake hydrogenase, diaphorase subunit	100	*C. saccharoperbutylacetonicum* ATCC 27021	HupC (74)	(Nakayama et al., 2008)

Protein ID	Predicted protein function	Average transcript abundance percentile (over all substrates)	Closest characterized functional homolog		
**Cphy_0090**	[FeFe] hydrogenase	94	*Thermoanaerobacterium saccharolyticum*	HfsD (60)	(Shaw et al., 2009)
**Cphy_0091**	RsbU regulatory phosphatase	95	*T. saccharolyticum*	HfsC(65)	(Shaw et al., 2009)
**Cphy_0092**	redox-sensing PAS-domain and [FeFe]hydrogenase	95	*T. saccharolyticum*	HfsB (60)	(Shaw et al., 2009)
**Cphy_0093**	hypothetical protein	86	*T. saccharolyticum*	HfsA (62)	(Shaw et al., 2009)
**Cphy_3801**	NADH-dependent [FeFe] hydrogenase, iron sulfur cluster containing subunit	94	*Thermoanaerobacter tengcongensis* MB4	HydC (67)	(Soboh et al., 2004)
			Thermotoga maritima MSB8	HydC (64)	(Schut & M. W. W. Adams, 2009)
**Cphy_3802**	histidine kinase	95	*T. tengcongensis* MB4	Baes4 (61)	(Soboh et al., 2004)
**Cphy_3803**	NADH-dependent [FeFe] hydrogenase, diaphorase subunit	93	*T. tengcongensis* MB4	HydD (76)	(Soboh et al., 2004)
			T. maritima MSB8	HyddD (59)	(Schut & M. W. W. Adams, 2009)
**Cphy_3804**	NADH-dependent [FeFe] hydrogenase, FMN- and NAD-binding diaphorase subunit	82	*T. tengcongensis* MB4	HydB (82)	(Soboh et al., 2004)
			T. maritima MSB8	HydB (77)	(Schut & M. W. W. Adams, 2009)

Protein ID	Predicted protein function	Average transcript abundance percentile (over all substrates)	Closest characterized functional homolog		
**Cphy_3805**	NADH-dependent [FeFe] hydrogenase, catalytic subunit	91	*T. tengcongensis* MB4	HydA (74)	(Soboh et al., 2004)
		**Energy production/Interconversion of redox equivalents**			
**Cphy_0211**	sodium-translocating ferredoxin-NAD oxidoreductase, RnfC NAD diaphorase subunit	95	*Acetobacterium woodii* DSM 1026	RnfC(61)	(Biegel et al., 2009; Biegel & Müller, 2010)
**Cphy_0212**	sodium-translocating ferredoxin-NAD oxidoreductase, FMN-binding RnfD subunit	87	*A. woodii* DSM 1026	RnfD (58)	(Biegel et al., 2009; Biegel & Müller, 2010)
**Cphy_0213**	sodium-translocating ferredoxin-NAD oxidoreductase, FMN-binding RnfG subunit	95	*A. woodii* DSM 1026	RnfG (47)	(Biegel et al., 2009; Biegel & Müller, 2010)
**Cphy_0214**	sodium-translocating ferredoxin-NAD oxidoreductase, integral membrane RnfE subunit	92	*A. woodii* DSM 1026	RnfE (59)	(Biegel et al., 2009; Biegel & Müller, 2010)
**Cphy_0215**	sodium-translocating ferredoxin-NAD oxidoreductase, integral membrane RnfA subunit	84	*A. woodii* DSM 1026	RnfA (74)	(Biegel et al., 2009; Biegel & Müller, 2010)
**Cphy_0216**	sodium-translocating ferredoxin-NAD oxidoreductase, iron-sulfur cluster binding RnfB subunit	97	*A. woodii* DSM 1026	RnfB (44)	(Biegel et al., 2009; Biegel & Müller, 2010)
		**Energy production/electrochemical gradient**			

Protein ID	Predicted protein function	Average transcript abundance percentile (over all substrates)	Closest characterized functional homolog		
**Cphy_3735**	atp synthase F1, epsilon subunit	85	*Clostridium paradoxum*	AtpC (43)	(Ferguson et al., 2006)
**Cphy_3736**	atp synthase F1, beta subunit	97	*C. paradoxum*	AtpD (85)	(Ferguson et al., 2006)
**Cphy_3737**	atp synthase F1, gamma subunit	94	*C. paradoxum*	AtpG(67)	(Ferguson et al., 2006)
**Cphy_3738**	atp synthase F1, alpha subunit	97	*C. paradoxum*	AtpA(86)	(Ferguson et al., 2006)
**Cphy_3739**	atp synthase F1, delta subunit	97	*C. paradoxum*	AtpH (58)	(Ferguson et al., 2006)
**Cphy_3740**	atp synthase F0, B subunit	97	*C. paradoxum*	AtpF (55)	(Ferguson et al., 2006)
**Cphy_3741**	atp synthase F0, C subunit	96	*C. paradoxum*	AtpE (78)	(Ferguson et al., 2006)
**Cphy_3742**	atp synthase F0, A subunit	97	*C. paradoxum*	AtpB (55)	(Ferguson et al., 2006)
**Citrate metabolism/energy production**					
**Cphy_2433**	oxaloacetate decarboxylase, alpha subunit	97	*Klebsiella pneumoniae*	OadA (53)	(Schwarz & Oesterhelt, 1985)
**Succinate metabolism/energy production**					
**Cphy_2434**	methyl-malonyl-CoA decarboxylase, sodium-translocating beta subunit	96	*Veillonella parvula*	MmdB (67)	(Huder & Dimroth, 1993)

Protein ID	Predicted protein function	Average transcript abundance percentile (over all substrates)	Closest characterized functional homolog		
**Cphy_2435**	methyl-malonyl-CoA decarboxylase, biotin-binding	95	*V. parvula*	MmdC (57)	(Huder & Dimroth, 1993)
**Cphy_2436**	methyl-malonyl-CoA decarboxylase, sodium-translocating delta subunit	90	*V. parvula*	MmdD (38)	(Huder & Dimroth, 1993)
**Cphy_2437**	methyl-malonyl-CoA decarboxylase, alpha subunit	95	*V. parvula*	MmdA (55)	(Huder & Dimroth, 1993)
		**Interconversion of reducing equivalents**			
**Cphy_2934**	NADH-dependent reduced ferredoxin:NADP oxidoreductase beta subunit	95	*Clostridium kluyveri* DSM 555	NfnB (84)	(Wang et al., 2010)
**Cphy_2935**	NADH-dependent reduced ferredoxin:NADP oxidoreductase alpha subunit	96	*C. kluyveri* DSM 555	NfnA (82)	(Wang et al., 2010)
		**Sodium homeostastis/unknown**			
**Cphy_3070**	sodium-transporting V-type atpase, D subunit	89	*Caloramator fervidus*	NtpD (73)	(Ubbink-Kok et al., 2006)
**Cphy_3071**	sodium-transporting V-type atpase, B subunit	94	*C. fervidus*	NtpB (88)	(Ubbink-Kok et al., 2006)
**Cphy_3072**	sodium-transporting V-type atpase, A subunit	92	*C. fervidus*	NtpA (81)	(Ubbink-Kok et al., 2006)
**Cphy_3073**	sodium-transporting V-type atpase, G subunit	93	*C. fervidus*	NtpG (65)	(Ubbink-Kok et al., 2006)
**Cphy_3074**	Sodium-transporting V-type atpase, C subunit	89	*C. fervidus*	NtpC(49)	(Ubbink-Kok et al., 2006)

Protein ID	Predicted protein function	Average transcript abundance percentile (over all substrates)	Closest characterized functional homolog		
**Cphy_3075**	sodium-transporting V-type atpase, E subunit	86	*C. fervidus*	NtpE(46)	(Ubbink-Kok et al., 2006)
**Cphy_3076**	sodium-transporting V-type atpase, K subunit	91	*C. fervidus*	NtpK (63)	(Ubbink-Kok et al., 2006)
**Cphy_3077**	sodium-transporting V-type atpase, I subunit	86	*C. fervidus*	NtpI (53)	(Ubbink-Kok et al., 2006)
**Cphy_3078**	sodium-transporting V-type atpase, F subunit	93	*C. fervidus*	NtpF (52)	(Ubbink-Kok et al., 2006)
**Cphy_3440**	V-type atpase, D subunit	74	*Ruminococcus obeum*	AtpD (82)	na
**Cphy_3441**	V-type atpase, B subunit	86	*R. obeum*	AtpB (90)	na
**Cphy_3442**	V-type atpase, A subunit	80	*R. obeum*	AtpA (85)	na
**Cphy_3443**	V-type atpase, E subunit	84	*R. obeum*	AtpE(62)	na
**Cphy_3444**	V-type atpase, F subunit	71	*R. obeum*	AtpF (88)	na
**Cphy_3445**	V-type atpase, K subunit	75	*R. obeum*	AtpC (65)	na
**Cphy_3446**	V-type atpase, I subunit	88	*R. obeum*	AtpI (67)	na

Protein ID	Predicted protein function	Average transcript abundance percentile (over all substrates)	Closest characterized functional homolog		
**Cphy_3447**	V-type atpase, C subunit	78	*R. obeum*	AtpC (62)	na

[a] *All percent similarity values were derived from pairwise global alignments of protein sequences generated using the algorithm of Needleman and Wunsch* (Needleman & Wunsch, 1970).
[b] Author and date

References :

Adams, M. W., Mortenson, L. E., & Chen, J. S. 1980. Hydrogenase. Biochim Biophys Acta 594: 105–76.

Alefounder, P. R., & Perham, R. N. 1989. Identification, molecular cloning and sequence analysis of a gene cluster encoding the class II fructose 1,6-bisphosphate aldolase, 3-phosphoglycerate kinase and a putative second glyceraldehyde 3-phosphate dehydrogenase of *Escherichia coli*. Molecular Microbiology 3: 723–32.

Amador-Noguez, D., Feng, X. J., Fan, J., Roquet, N., Rabitz, H., & Rabinowitz, J. D. 2010. Systems-level metabolic flux profiling elucidates a complete, bifurcated tricarboxylic acid cycle in *Clostridium acetobutylicum*. Journal of Bacteriology 192: 4452–61.

Asanuma, N., & Hino, T. 2006. Presence of NAD+-specific glyceraldehyde-3-phosphate dehydrogenase and CcpA-dependent transcription of its gene in the ruminal bacterium *Streptococcus bovis*. FEMS Microbiology Letters 257: 17–23.

Asanuma, Narito, Kanada, K., Arai, Y., Yoshizawa, K., Ichikawa, T., & Hino, Tsuneo. 2010. Molecular characterization and significance of phosphoenolpyruvate carboxykinase in a ruminal bacterium, *Streptococcus bovis*. The Journal of General and Applied Microbiology 56: 121–127.

Bae, E., & Phillips, G. N., Jr. 2004. Structures and analysis of highly homologous psychrophilic, mesophilic, and thermophilic adenylate kinases. The Journal of Biological Chemistry 279: 28202–28208.

Belouski, E., Watson, D., & Bennett, G. 1998. Cloning, sequence, and expression of the phosphofructokinase gene of *Clostridium acetobutylicum* ATCC 824 in Escherichia coli. Current Microbiology 37: 17–22.

Biegel, E., & Müller, V. 2010. Bacterial Na+-translocating ferredoxin:NAD+ oxidoreductase. Proceedings of the National Academy of Sciences of the United States of America 107: 18138–18142. Biegel, E., Schmidt, S., González, J. M., & Müller, V. 2011. Biochemistry, evolution and physiological function of the Rnf complex, a novel ion-motive electron transport complex in prokaryotes. Cellular and Molecular Life Sciences: CMLS 68: 613–634.

Biegel, E., Schmidt, S., & Müller, V. 2009. Genetic, immunological and biochemical evidence for a Rnf complex in the acetogen *Acetobacterium woodii*. Environmental Microbiology 11: 1438–1443.

Buckel, W. 2001. Sodium ion-translocating decarboxylases. Biochimica Et Biophysica Acta 1505: 15–27.

Calusinska, M., Happe, T., Joris, B., & Wilmotte, A. 2010. The surprising diversity of clostridial hydrogenases: a comparative genomic perspective. Microbiology (Reading, England) 156: 1575–1588.

Ferguson, S. A., Keis, S., & Cook, G. M. 2006. Biochemical and molecular characterization of a Na+-translocating F1Fo-ATPase from the thermoalkaliphilic bacterium *Clostridium paradoxum*. Journal of Bacteriology 188: 5045–5054.

Huder, J. B., & Dimroth, P. 1993. Sequence of the sodium ion pump methylmalonyl-CoA decarboxylase from *Veillonella parvula*. The Journal of Biological Chemistry 268: 24564–71.

Kabir, M. M., & Shimizu, K. 2004. Metabolic regulation analysis of icd-gene knockout *Escherichia coli* based on 2D electrophoresis with MALDI-TOF mass spectrometry and enzyme activity measurements. Applied Microbiology and Biotechnology 65: 84–96. Retrieved April 13, 2012,

Kessler, D., Leibrecht, I., & Knappe, J. 1991. Pyruvate-formate-lyase-deactivase and acetyl-CoA reductase activities of *Escherichia coli* reside on a polymeric protein particle encoded by adhE. FEBS Letters 281: 59–63.

Latimer, M. T., & Ferry, J. G. 1993. Cloning, sequence analysis, and hyperexpression of the genes encoding phosphotransacetylase and acetate kinase from *Methanosarcina thermophila*. Journal of Bacteriology 175: 6822–6829.

Leibig, M., Liebeke, M., Mader, D., Lalk, M., Peschel, A., & Götz, F. 2011. Pyruvate formate lyase acts as a formate supplier for metabolic processes during anaerobiosis in *Staphylococcus aureus*. Journal of Bacteriology 193: 952–962.

Leyva-Vazquez, M. A., & Setlow, P. 1994. Cloning and nucleotide sequences of the genes encoding triose phosphate isomerase, phosphoglycerate mutase, and enolase from *Bacillus subtilis*. Journal of Bacteriology 176: 3903–10.

Llanos, R. M., Hillier, A. J., & Davidson, B. E. 1992. Cloning, nucleotide sequence, expression, and chromosomal location of ldh, the gene encoding L-(+)-lactate dehydrogenase, from *Lactococcus lactis*. Journal of Bacteriology 174: 6956–6964.

Meinecke, B., Bertram, J., & Gottschalk, G. 1989. Purification and characterization of the pyruvate-ferredoxin oxidoreductase from *Clostridium acetobutylicum*. Archives of Microbiology 152: 244–250.

Mertens, E. 1993. ATP versus pyrophosphate: glycolysis revisited in parasitic protists. Parasitology Today (Personal Ed.) 9: 122–126.

Mitchell, C., Morris, P. W., Lum, L., Spiegelman, G., & Vary, J. C. 1992. The amino acid sequence of a *Bacillus subtilis* phosphoprotein that matches an orfY-tsr coding sequence. Molecular Microbiology 6: 1345–1349.

Muñoz, M., Le Borgne, S., Bolívar, F., & Valle, F. 1997. Molecular cloning of the gene that codes for the pyruvate kinase of *Bacillus subtilis*: primary characterization of a strain carrying this gene insertionally inactivated. Revista Latinoamericana De Microbiología 39: 129–140.

Nakayama, S., Kosaka, T., Hirakawa, H., Matsuura, K., Yoshino, S., & Furukawa, K. 2008. Metabolic engineering for solvent productivity by downregulation of the hydrogenase gene cluster hupCBA in *Clostridium saccharoperbutylacetonicum* strain N1-4. Applied Microbiology and Biotechnology 78: 483–493.

Needleman, S., & Wunsch, C. 1970. A general method applicable to the search for similarities in the amino acid sequence of two proteins. Journal of Molecular Biology 48: 443–453.

Ng, W. V., Kennedy, S. P., Mahairas, G. G., Berquist, B., Pan, M., Shukla, H. D., Lasky, S. R., Baliga, N. S., Thorsson, V., Sbrogna, J., Swartzell, S., Weir, D., Hall, J., Dahl, T. A., Welti, R., et al. 2000. Genome sequence of *Halobacterium* species NRC-1. Proceedings of the National Academy of Sciences of the United States of America 97: 12176–12181.

Ohara, O., Dorit, R. L., & Gilbert, W. 1989. Direct genomic sequencing of bacterial DNA: the pyruvate kinase I gene of *Escherichia coli*. Proceedings of the National Academy of Sciences of the United States of America 86: 6883–6887.

Pichersky, E., Gottlieb, L. D., & Hess, J. F. 1984. Nucleotide sequence of the triose phosphate isomerase gene of *Escherichia coli*. Molecular & General Genetics: MGG 195: 314–320.

Plaga, W., Lottspeich, F., & Oesterhelt, D. 1992. Improved purification, crystallization and primary structure of pyruvate:ferredoxin oxidoreductase from *Halobacterium halobium*. European Journal of Biochemistry / FEBS 205: 391–397.

Pocalyko, D. J., Carroll, L. J., Martin, B. M., Babbitt, P. C., & Dunaway-Mariano, D. 1990. Analysis of sequence homologies in plant and bacterial pyruvate phosphate dikinase, enzyme I of the bacterial phosphoenolpyruvate: sugar phosphotransferase system and other PEP-utilizing enzymes. Identification of potential catalytic and regulatory motifs. Biochemistry 29: 10757–10765.

Ponce, E., Flores, N., Martinez, A., Valle, F., & Bolívar, F. 1995. Cloning of the two pyruvate kinase isoenzyme structural genes from *Escherichia coli*: the relative roles of these enzymes in pyruvate biosynthesis. Journal of Bacteriology 177: 5719–5722.

Prasad, C., Diesterhaft, M., & Freese, E. 1972. Initiation of spore germination in glycolytic mutants of *Bacillus subtilis*. Journal of Bacteriology 110: 321–328.

Ren, Z., Ward, T., Logan, B., & Regan, J. 2007. Characterization of the cellulolytic and hydrogen-producing activities of six mesophilic Clostridium species. Journal of Applied Microbiology 103: 2258–2266.

Reshetnikov, A. S., Rozova, O. N., Khmelenina, V. N., Mustakhimov, I. I., Beschastny, A. P., Murrell, J. C., & Trotsenko, Y. A. 2008. Characterization of the pyrophosphate-dependent 6-phosphofructokinase from *Methylococcus capsulatus* Bath. FEMS Microbiology Letters 288: 202–210.

Rödel, W., Plaga, W., Frank, R., & Knappe, J. 1988. Primary structures of *Escherichia coli* pyruvate formate-lyase and pyruvate-formate-lyase-activating enzyme deduced from the DNA nucleotide sequences. European Journal of Biochemistry / FEBS 177: 153–158.

Schreiber, W., & Durre, P. 1999. The glyceraldehyde-3-phosphate dehydrogenase of *Clostridium acetobutylicum*: isolation and purification of the enzyme, and sequencing and localization of the gap gene within a cluster of other glycolytic genes. Microbiology 145 (Pt 8): 1839–47.

Schut, G., & Adams, M. 2009. The iron-hydrogenase of *Thermotoga maritima* utilizes ferredoxin and NADH synergistically: a new perspective on anaerobic hydrogen production. Journal of Bacteriology 191: 4451–4457.

Schut, G. J., & Adams, M. W. W. 2009. The iron-hydrogenase of *Thermotoga maritima* utilizes ferredoxin and NADH synergistically: a new perspective on anaerobic hydrogen production. Journal of Bacteriology 191: 4451–4457.

Schwarz, E., & Oesterhelt, D. 1985. Cloning and expression of *Klebsiella pneumoniae* genes coding for citrate transport and fermentation. The EMBO Journal 4: 1599–1603.

Shaw, A., Hogsett, D., & Lynd, L. 2009. Identification of the [FeFe]-hydrogenase responsible for hydrogen generation in *Thermoanaerobacterium saccharolyticum* and demonstration of increased ethanol yield via hydrogenase knockout. Journal of Bacteriology 191: 6457–6464.

Skarlatos, P., & Dahl, M. 1998. The glucose kinase of *Bacillus subtilis*. Journal of Bacteriology 180: 3222–3226.

Soboh, B., Linder, D., & Hedderich, R. 2004. A multisubunit membrane-bound [NiFe] hydrogenase and an NADH-dependent Fe-only hydrogenase in the fermenting bacterium *Thermoanaerobacter tengcongensis*. Microbiology (Reading, England) 150: 2451–2463.

Thauer, R. K., Jungermann, K., & Decker, K. 1977. Energy conservation in chemotrophic anaerobic bacteria. Bacteriological Reviews 41: 100–80.

Tolonen, A., Haas, W., Chilaka, A., Aach, J., Gygi, S., & Church, G. 2011. Proteome-wide systems analysis of a cellulosic biofuel-producing microbe. Molecular Systems Biology 7: 461.

Ubbink-Kok, T., Nijland, J., Slotboom, D., & Lolkema, J. 2006. The ntp operon encoding the Na+ V-ATPase of the thermophile *Caloramator fervidus*. Archives of Microbiology 186: 513–517.

Walter, K. A., Bennett, G. N., & Papoutsakis, E. T. 1992. Molecular characterization of two *Clostridium acetobutylicum* ATCC 824 butanol dehydrogenase isozyme genes. Journal of Bacteriology 174: 7149–58.

Wang, S., Huang, H., Moll, J., & Thauer, R. 2010. NADP+ reduction with reduced ferredoxin and NADP+ reduction with NADH are coupled via an electron-bifurcating enzyme complex in *Clostridium kluyveri*. Journal of Bacteriology 192: 5115–5123

Warnick, T., Methé, B., & Leschine, S. 2002. *Clostridium phytofermentans* sp. nov., a cellulolytic mesophile from forest soil. International Journal of Systematic and Evolutionary Microbiology 52: 1155–1160.

Winzer, K., Lorenz, K., & Dürre, P. 1997. Acetate kinase from Clostridium acetobutylicum: a highly specific enzyme that is actively transcribed during acidogenesis and solventogenesis. Microbiology (Reading, England) 143 (Pt 10): 3279–3286.

Youngleson, J. S., Jones, W. A., Jones, D. T., & Woods, D. R. 1989. Molecular analysis and nucleotide sequence of the adh1 gene encoding an NADPH-dependent butanol dehydrogenase in the Gram-positive anaerobe *Clostridium acetobutylicum*. Gene 78: 355–64.
